# AlphaCRV: a pipeline for identifying accurate binder topologies in mass-modeling with AlphaFold

**DOI:** 10.1093/bioadv/vbae131

**Published:** 2024-09-06

**Authors:** Francisco J Guzmán-Vega, Stefan T Arold

**Affiliations:** Biological and Environmental Science and Engineering Division, Computational Biology Research Center, King Abdullah University of Science and Technology, Thuwal 23955-6900, Kingdom of Saudi Arabia; Biological and Environmental Science and Engineering Division, Computational Biology Research Center, King Abdullah University of Science and Technology, Thuwal 23955-6900, Kingdom of Saudi Arabia

## Abstract

**Motivation:**

The speed and accuracy of deep learning-based structure prediction algorithms make it now possible to perform in silico “pull-downs” to identify protein–protein interactions on a proteome-wide scale. However, on such a large scale, existing scoring algorithms are often insufficient to discriminate biologically relevant interactions from false positives.

**Results:**

Here, we introduce AlphaCRV, a Python package that helps identify correct interactors in a one-against-many AlphaFold screen by clustering, ranking, and visualizing conserved binding topologies, based on protein sequence and fold.

**Availability and implementation:**

AlphaCRV is a Python package for Linux, freely available at https://github.com/strubelab/AlphaCRV

## 1 Introduction

Protein–protein interactions are the foundation of most biological processes. With the advent of increasingly efficient and reliable computational methods, such as AlphaFold-Multimer (Evans *et al.* 2022), AlphaFold3 (Abramson *et al.* 2024), and other deep learning methods, rapid screening of protein–protein interactions has become feasible. This technological leap has paved the way for *in silico* identification and characterization of previously unknown complexes (Humphreys *et al.* 2021, Burke *et al.* 2023, O’Reilly *et al.* 2023). Recent enhancements in algorithmic efficiency have further accelerated modeling speed, enabling proteome–scale interaction predictions as a computational alternative to high-throughput experimental investigations (Mirdita *et al.* 2022, Wallner 2023, Yu *et al.* 2023, Abramson *et al.* 2024, Ahdritz *et al.* 2024). However, the accuracy of protein complex predictions still falls short when compared to monomeric structures (Bryant 2023) and accurately identifying correct binding partners from among thousands of predictions remains a significant challenge.

Active efforts are underway to develop computable quality scores for evaluating multimeric models (Malhotra *et al.* 2021, Bryant et al. 2022a,b, Zhao *et al.* 2024). Yet, at proteome levels, these scores (or their combinations) currently lack the selectivity needed to pinpoint the correct solutions, leading to a high number of false positives and rendering experimental verification impractical.

To address this challenge, we have developed AlphaCRV (an acronym for “AlphaFold Cluster, Rank, and Visualize”). AlphaCRV is designed to help identify true positives in a large-scale AlphaFold screen of a bait protein against proteomes. The rationale behind AlphaCRV is that the bait protein should bind in a similar manner to similar ligands with similarly good scores. Accordingly, AlphaCRV clusters the top models based on sequence and fold to identify structurally similar binders, ranks them according to cluster size and topological similarity, and generates files for PyMOL visualization. True interactions can then be distinguished from false positives as those that form large clusters with a low root mean square deviation (RMSD) and high individual scores. We show that AlphaCRV successfully identifies biologically relevant binders in screens of a bait protein against all 43 595 proteins of the rice proteome.

## 2 Implementation

AlphaCRV is written in Python, with a command-line interface that allows the user to specify different parameters for the clustering process. It also allows the user to rank the resulting clusters and produce PyMOL sessions of the top clusters for easy visualization. The program is separated into two steps: (i) clustering and (ii) ranking and visualization.

### 2.1 Clustering

**Figure 1.**
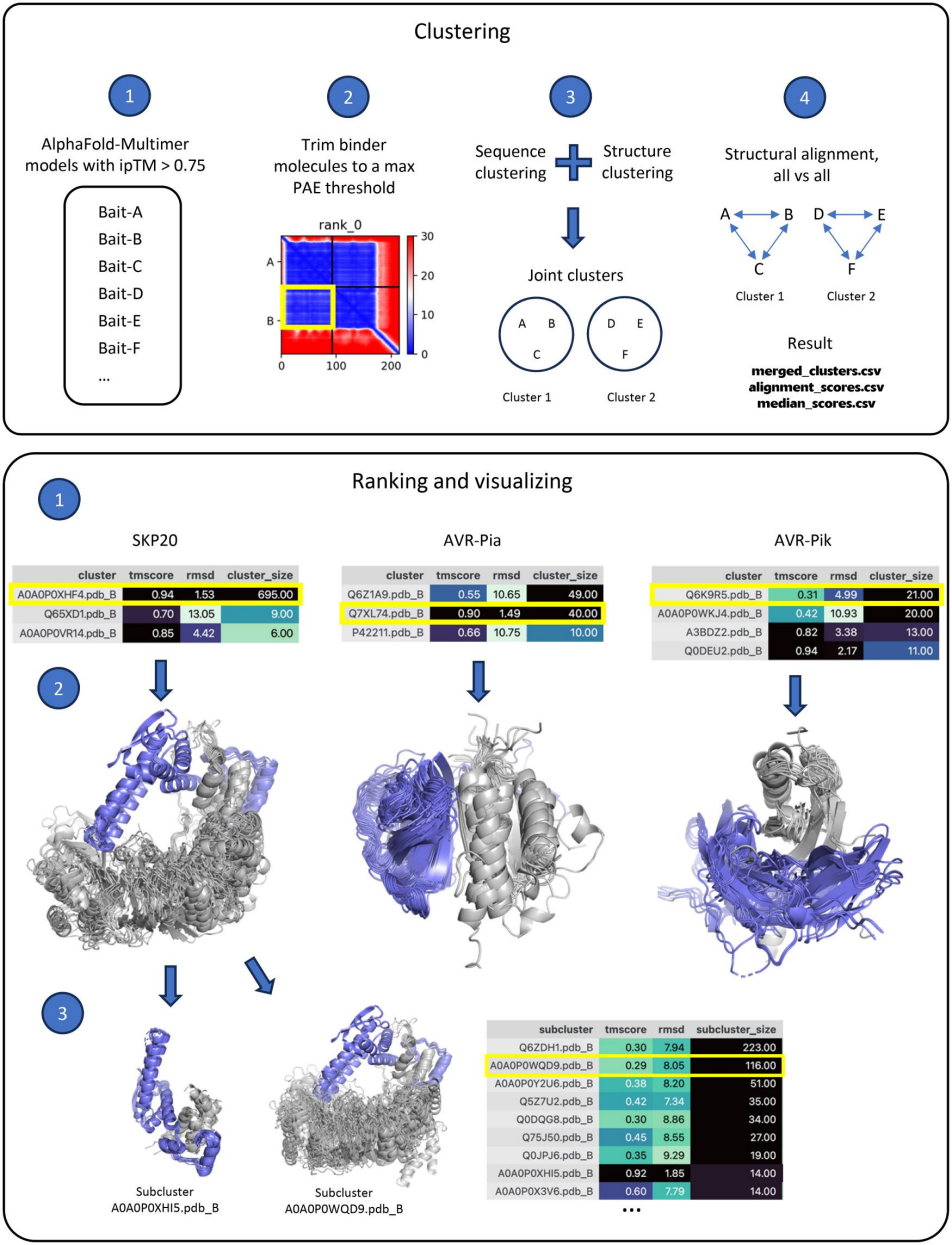
The AlphaCRV pipeline. The pipeline is divided into two stages: Clustering (upper panel) and Ranking and Visualization (lower panel). Upper panel (2) shows the PAE matrix, colored from blue to red according to the estimated error in Å. *X*-axis shows residue numbers, and *Y*-axis shows segments for proteins A and B. The yellow box encircles the off-diagonal field indicating a possible interaction, which is used for trimming the ligands prior to clustering. Lower panel (1): AlphaCRV was applied to a proteome-wide *in silico* interaction screen of three proteins with different MSA depths: SKP1-like protein 20 (left), AVR-Pia (center), and AVR-Pik (right). The tables show the top-ranked clusters sorted by cluster size. The clusters containing the homologs of the experimentally confirmed targets are highlighted in yellow. The clusters are labeled according to the UniProt code of the cluster representative with the extension .pdb_B. These clusters can be identified by the combination of a large cluster size and low RMSD of the cluster representative. Lower panel (2): PyMOL session produced at the Visualization step. The bait proteins are colored blue, and the (PAE-trimmed) rice interactors are gray. Lower panel (3): Subclusters identified within the top cluster of SKP20. The subcluster containing the experimentally confirmed target is highlighted in yellow. The full analysis of these case studies is provided in the Supplementary Note and in the GitHub repository (see Availability and implementation section).

AlphaCRV takes as input a directory containing the ranking_debug.json, .pdb, and .pkl files for the best AlphaFold-Multimer prediction of each possible complex. In principle, these models may include different kinds of dimeric interactions including transient interactions, or interactions with disordered peptides or short linear motifs. The inclusion of interactions in the AlphaCRV analysis depends on their interface-predicted TM (ipTM) score and the ipTM threshold set by the user. The specific approach for obtaining these models will vary depending on the computational resources available, the implementation of AlphaFold-Multimer used, and any other strategies employed to optimize model quality (Bret *et al.* 2024, Lee *et al.* 2024). It will be up to the user to select the appropriate strategy for model generation and provide the list of AlphaFold-Multimer models to be used for clustering with AlphaCRV. For our test cases, we generated the models using our in-house AlphaFold-Multimer implementation (Guzmán-Vega and Arold 2024). The models may also be produced with other tools such as AlphaPulldown (Yu *et al.* 2023). AlphaFold 3 also produces the ipTM and Predicted Aligned Error (PAE) scores used by AlphaCRV (although with a different output format as AlphaFold2), but the server-only implementation of AlphaFold 3 currently prevents its use to generate input models on a proteome scale. AlphaCRV first trims these complexes to only include the interacting domains of the bait and target proteins. The resulting complexes are then clustered based on either the protein fold or the sequence of the target proteins. Finally, AlphaCRV groups the sequence and structure clusters that share common elements into joint clusters (Fig. 1, upper panel). The combination of both sequence and structure clustering makes AlphaCRV more sensitive to finding proteins with remote homologies that might otherwise be missed by either method alone.

In detail, these steps are as follows:

1. Model Selection Based on Quality Scores: The process begins by selecting models with an ipTM score (Zhang and Skolnick 2004, Evans *et al.* 2022) equal to or greater than the user-defined threshold (default value is 0.75). This step ensures that only models with high-quality interface predictions are retained.
2. Trimming of PDB Models and Sequences: The Biskit Python library (Grünberg *et al.* 2007) is used to trim the PDB models and sequences based on a PAE (Jumper *et al.* 2021, Evans *et al.* 2022) threshold. This step retains only the protein domains/regions of the target that have a confident positioning relative to the bait molecule.
3. Sequence and Structural Clustering: Sequence clustering is performed on the trimmed sequences using MMseqs2 (Steinegger and Söding 2017), and structural clustering is carried out on the trimmed structures using Foldseek (Barrio-Hernandez *et al.* 2023). These algorithms were chosen for their speed and sensitivity in clustering the sequences and structures of full proteomes. Sequence and structure clusters containing common target proteins are then merged into joint clusters that capture both sequence and structure relationships within the targets. Different structure clusters may be merged if they have elements with related sequences between them, resulting in substantially larger clusters than could be achieved by either method alone.
4. Identification of the Best Representative Structure for Each Joint Cluster: A pairwise all-vs-all structural alignment of the members is performed using US-align (Zhang *et al.* 2022). The best representative for each cluster is defined as the one with the lowest median RMSD when aligned to all other cluster members.

The clustering stage outputs csv files that contain a list of complexes with their corresponding quality scores from AlphaFold, as well as their sequence and structure cluster, separated and merged, and the median RMSDs and TM-scores of each cluster member when aligned to all other members of the same merged cluster (see Supplementary Note for more details and examples). This information is used as input for the next step to select the best clusters (with most consistent structures) and best cluster representatives.

### 2.2 Ranking and visualization

The second stage of the pipeline ranks the clusters based on several user-defined criteria and produces PyMOL sessions for visualization. Optionally, this stage can also be used to identify subclusters within the top clusters. This could be insightful for large clusters that contain different binding modes for the same target topology, or when different targets share a common binding domain.

The individual steps performed by this stage are (Fig. 1, bottom panel):

1. Cluster Ranking: The clusters from the previous stage are ranked based on several factors: The size of the cluster, the median TM-score, the median RMSD, and the alignment coverage of the cluster representative.
2. Creation of PyMOL Sessions: The PDB files of each of the top clusters are copied to individual directories, and PyMOL sessions are created for each.
3. Optional Subclustering: Optionally, Foldseek can be used again to perform structural clustering within the top clusters, and the results can be saved for further analysis.

Starting from thousands of complex predictions with similarly high binding scores, a successful AlphaCRV run identifies a small number of bait-target clusters (usually fewer than five) with consistent binding topology and high scores. Furthermore, the process generates .pse files for convenient examination of these clusters with PyMOL. A detailed example of the analysis workflow is available on the GitHub repository (see Availability and implementation section) as Jupyter notebooks, which are also included in the Supplementary Note.

## 3 Test cases

For each test case, AlphaCRV requires a proteome-scale bait-target AlphaFold prediction. The time and resources required for the generation and storage of many thousands of complexes per bait limited our evaluation of AlphaFold to six bait-proteome cases.

We first applied AlphaCRV to an interaction screen of three bait proteins against the 43 595 proteins of the rice reference proteome (*O. sativa subsp. japonica*). Using an in-house implementation of AlphaFold-Multimer (Guzmán-Vega and Arold 2024) we predicted two complexes for each bait–target pair (87 190 complexes per bait). As first bait we chose the SKP1-like protein 20 (SKP20; UniProt ID Q651E8), a component of SKIP-cullin—F box (SCF) E3 ubiquitin ligase complexes. SKP20 produces a deep multiple sequence alignment (MSA) and, as a linker between cullins and F-box proteins, has many potential ligands. The other two baits were fungal effector proteins, AVR-Pia (B9WZW9), and AVR-Pik (C4B8B8), both of which have few or no known sequence homologs and are expected to have only few interacting rice proteins (Fig. 1).

For SKP20 the ipTM (Evans *et al.* 2022), pDockQ (Bryant *et al.* 2022a), and interface pLDDT (ipLDDT) (Jumper *et al.* 2021, Evans *et al.* 2022) scores ranked the known F-box complex (D3, UniProt ID Q5VMP0) in 3rd, 1st, and 90th place, respectively, out of 950 complexes with an ipTM score above 0.75 (Supplementary Table 1). AlphaCRV identified a major cluster with 695 targets, high TM-score and low RMSD, which contains D3. The second cluster groups targets with remote similarity to F-box proteins, whereas the third cluster features cullin-like targets. Subclustering of the major cluster generated two structurally distinct clusters with F-box-like interfaces (Fig. 1, Ranking and visualizing; 2,3). Thus, out of 43 595 potential ligands, AlphaCRV identified three (or five) clusters containing plausible F-box and cullin interactors.

For AVR-Pia and AVR-Pik, the experimentally confirmed targets (HMA domains) were only ranked 65 to 520 by ipTM, pDockQ, and ipLDDT (Supplementary Tables 2 and 3). Conversely, AlphaCRV consistently identified the experimental binder and the correct topology as one of the top three largest clusters. In each case, the cluster with the experimentally confirmed topology was the one with the best combination of large cluster size and low RMSD, but not the one containing the member with the best ipTM, pDockQ, and ipLDDT scores (Supplementary Figs 1 and 2).

 In the tested cases of SKP20, AVR-Pia, and AVR-Pik, the known biological binders have 5 (F-box), 15, and 16 sequences in the rice proteome with more than 25% identity, respectively. This allowed AlphaCRV to successfully identify clusters with the known binders in each case. However, due to its strategy of clustering homologous interactions, AlphaCRV may fail if the true binder has no or only very few homologs in the screened proteome.

To assess this limitation, we tested AlphaCRV on three bait screens against the 4 400 protein E. coli proteome. AlphaCRV successfully placed the experimentally confirmed homodimeric complex of UspA (UniProt ID P0AED0) into the largest cluster (Supplementary Fig. 3). This cluster contained five elements, namely UspA and its paralogs UspC, D, F, and G, suggesting heterodimers can form within this protein family.

However, AlphaCRV failed for the bait proteins TRCF and LPTE (UniProt IDs P30958 and P0ADC1). Although the complexes with the true binders of these proteins (P0A698 for TRCF and P31554 for LPTE) were modeled with high quality by AlphaFold (ipTM > 0.75), AlphaCRV did not assign them to any cluster because the true binders lack homologs in E. coli (Supplementary Fig. 3). These test cases show that in large-scale searches, AlphaCRV may help identifying different classes of ligands for proteins with many targets (case of SKP20), or help finding the true binder for baits with shallow MSAs (cases of fungal effectors AVR-Pia and AVR-Pik). However, if the true target has fewer than four homologs in the dataset, AlphaCRV may fail. This issue could be addressed by pooling together proteomes from related species to increase the number of homologs to the true binder.

If the failure of AlphaCRV to identify promising clusters stems from an absence of high-ipTM scored targets (e.g. for transient interactions or complexes that are less well predicted and scored by AlphaFold-Multimer), then the ipTM score requirement may be lowered. Alternatively, complex prediction by AlphaFold-Multimer may be enhanced, e.g. by testing different domains or fragments of the interacting proteins (Bret *et al.* 2024, Lee *et al.* 2024), or by enforcing a dropout at inference time to increase sampling of different conformations (Johansson-Åkhe and Wallner 2022). Similarly, there are strategies to speed up the generation of protein complex models as input for AlphaCRV. These approaches include separating the CPU- and GPU-intensive steps of AlphaFold-Multimer by precomputing the MSAs and MSA-based features (Yu *et al.* 2023, Guzmán-Vega and Arold 2024), using faster MSA algorithms (Steinegger and Söding 2017, Mirdita *et al.* 2022), or using faster 3D modeling methods such as ESMFold for a prescreening step (Lin *et al.* 2023). Another strategy would be to only calculate complex models for “likely” partners, e.g. proteins present the in same subcellular localization, or identified by other types of high-throughput network or binding analyses.

AlphaCRV is the first method to use clustering to identify biologically relevant complexes at a proteome-scale. This approach is different from docking or interface-scoring algorithms, precluding a direct comparison with existing tools. In fact, the clustering strategy of AlphaCRV is based on the observation that there is currently no single score that can confidently identify true from false positives. Hence, the best use of AlphaCRV is as a tool to select a handful of promising clusters from an initial pool of tens to hundreds of thousands of potential complexes and to prepare them for convenient manual inspection. However, AlphaCRV also provides useful metrics to assess the likelihood of the clusters containing true binders. These metrics include the cluster size (the larger the better) and the TM and RMSD scores (the lower the better). Furthermore, the AlphaCRV result files contain the ipTM and ipTM+pTM scores from AlphaFold-Multimer (in 'output_directory/merged_clusters/merged_clusters.csv'; see Supplementary Note). These scores can be used to further evaluate the AlphaCRV clusters and rank the models within each of them, jointly with manual inspection of the complexes using the PyMOL files provided. Any additional “orthogonal” information supporting the functional importance of the predicted binding regions in the cluster would be useful to consider, such as conservation scores.

We measured the run times of AlphaCRV in three replicates for the six test cases on a Linux-based machine with 8 CPU cores and 8 GB RAM (32 GB RAM for the alphacrv-rank step with SKP20) (Supplementary Fig. 4). The clustering and alignment of complexes for SKP20, Avr-Pia, and Avr-Pik required a mean run time of 110, 3, and 2 min, respectively, while the same step on the complexes for uspA, TRCF and LPTE, required a mean run time of 0.5, 1, and 2 min. The most computationally expensive step is the all-vs-all alignment of the cluster members to find the best cluster representative. This step increases the runtime exponentially with the number of complexes in the clusters (the largest clusters from the SKP20, Avr-Pia, and Avr-Pik analyses have 695, 49, and 21 members, respectively). The --cpus parameter can be used to linearly reduce the structural alignment times by distributing their computation over multiple CPUs, enabling inclusion of a larger number of initial complexes by lowering the --iptm_threshold parameter.

## Supplementary Material

vbae131_Supplementary_Data

## Data Availability

AlphaCRV is available for download in our GitHub repository (https://github.com/strubelab/AlphaCRV). It can be easily installed on any Linux machine through a conda environment. Detailed documentation and instructions for installation, usage, and examples are provided in the repository.
