# Supplementary material for: AlphaCRV: a pipeline for identifying accurate binder topologies in mass-modeling with AlphaFold
Source: Bioinform Adv. 2024 Sep 6;4(1):vbae131. doi: 10.1093/bioadv/vbae131 (PMC11405088; doi:10.1093/bioadv/vbae131)
Supplement: vbae131_Supplementary_Data [file vbae131_supplementary_data.zip › vbae131_Supplementary_Data/Supplementary_Data_2024-08.docx]

| **Rank by ipTM score** | | | | |
| --- | --- | --- | --- | --- |
| rank | complex | ipTM | pDockQ | interface_pLDDT |
| 1 | 8IF6-1_Q6Z1A9-1 | 0.9033 | 0.7391 | 95.0026 |
| 2 | 8IF6-1_Q7XSL8-1 | 0.9029 | 0.7380 | 87.3810 |
| 3 | 8IF6-1_Q5VMP0-1* | 0.8992 | 0.7401 | 92.4547 |
| 8 | 8IF6-1_A0A0P0Y6A8-1 | 0.8976 | 0.7380 | 89.2389 |
| 339 | 8IF6-1_Q8RZQ3-1 | 0.8671 | 0.7390 | 95.0096 |
| 399 | 8IF6-1_A0A0N7KEW0-1 | 0.8625 | 0.7361 | 87.0794 |
| 554 | 8IF6-1_Q69X07-1 | 0.8473 | 0.7288 | 80.7505 |
| **Rank by pDockQ score** | | | | |
| rank | complex | ipTM | pDockQ | interface_pLDDT |
| 1 | 8IF6-1_Q5VMP0-1* | 0.8992 | 0.7401 | 92.4547 |
| 2 | 8IF6-1_Q0E1Z5-1 | 0.8911 | 0.7398 | 93.7280 |
| 3 | 8IF6-1_Q7XVM8-1 | 0.8888 | 0.7398 | 94.9729 |
| 24 | 8IF6-1_Q8RZQ3-1 | 0.8671 | 0.7390 | 95.0096 |
| 128 | 8IF6-1_A0A0P0Y6A8-1 | 0.8976 | 0.7380 | 89.2389 |
| 363 | 8IF6-1_A0A0N7KEW0-1 | 0.8625 | 0.7361 | 87.0794 |
| 675 | 8IF6-1_Q69X07-1 | 0.8473 | 0.7288 | 80.7505 |
| **Rank by Interface pLDDT score** | | | | |
| rank | complex | ipTM | pDockQ | interface_pLDDT |
| 1 | 8IF6-1_Q60EH3-1 | 0.7568 | 0.4524 | 95.9317 |
| 2 | 8IF6-1_A0A0P0XGV2-1 | 0.8592 | 0.7391 | 95.5453 |
| 3 | 8IF6-1_Q7FAH1-1 | 0.8638 | 0.7392 | 95.3636 |
| 5 | 8IF6-1_Q8RZQ3-1 | 0.8671 | 0.7390 | 95.0096 |
| 90 | 8IF6-1_Q5VMP0-1* | 0.8992 | 0.7401 | 92.4547 |
| 308 | 8IF6-1_A0A0P0Y6A8-1 | 0.8976 | 0.7380 | 89.2389 |
| 462 | 8IF6-1_A0A0N7KEW0-1 | 0.8625 | 0.7361 | 87.0794 |
| 742 | 8IF6-1_Q69X07-1 | 0.8473 | 0.7288 | 80.7505 |

**Supplementary Table 1.** Ranking of complexes of SKP20 with true binder and homologous sequences of the true binder in rice. The top scored complexes overall that are not homologs are shaded grey. 8IF6 is the PDB ID of the experimentally known complex from which the SKP20 sequence was obtained. * Complex with full chain of true binder.

| **Rank by ipTM score** | | | | |
| --- | --- | --- | --- | --- |
| rank | complex | ipTM | pDockQ | interface_pLDDT |
| 1 | 6Q76-1_Q0IT61-1 | 0.9573 | 0.7082 | 89.0398 |
| 2 | 6Q76-1_Q851F1-1 | 0.9550 | 0.7291 | 90.4324 |
| 3 | 6Q76-1_Q653R0-1 | 0.9530 | 0.7294 | 93.4237 |
| 159 | 6Q76-1_Q6EPT2-1 | 0.9138 | 0.7220 | 96.2785 |
| 245 | 6Q76-1_Q6EPT4-1 | 0.9038 | 0.7172 | 94.1533 |
| 343 | 6Q76-1_Q2QSQ7-1 | 0.8921 | 0.7202 | 94.3160 |
| 388 | 6Q76-1_Q0J314-1 | 0.8863 | 0.7136 | 94.8359 |
| 440 | 6Q76-1_6Q76A-1* | 0.8808 | 0.7250 | 95.7609 |
| 488 | 6Q76-1_Q8S5W0-1 | 0.8757 | 0.7208 | 93.9732 |
| 554 | 6Q76-1_Q6YY34-1 | 0.8673 | 0.7156 | 93.7823 |
| 582 | 6Q76-1_Q7XJV3-1 | 0.8622 | 0.7171 | 94.1851 |
| 592 | 6Q76-1_Q6YY33-1 | 0.8609 | 0.7127 | 92.9624 |
| 624 | 6Q76-1_A0A0N7KFK3-1 | 0.8571 | 0.7128 | 93.3532 |
| 652 | 6Q76-1_A0A0P0VKX7-1 | 0.8541 | 0.7129 | 91.9085 |
| 724 | 6Q76-1_A0A0P0WB87-1 | 0.8431 | 0.6985 | 89.4861 |
| 835 | 6Q76-1_Q6YY31-1 | 0.8251 | 0.7001 | 89.5552 |
| 879 | 6Q76-1_Q0JCK8-1 | 0.8183 | 0.7016 | 91.1076 |
| 948 | 6Q76-1_Q7XJV0-1 | 0.8064 | 0.6999 | 89.1890 |
| 1438 | 6Q76-1_E9KPB5-1** | 0.6884 | 0.5947 | 74.9171 |
| **Rank by pDockQ score** | | | | |
| rank | complex | ipTM | pDockQ | interface_pLDDT |
| 1 | 6Q76-1_A0A0P0X879-1 | 0.9117 | 0.7407 | 91.2456 |
| 2 | 6Q76-1_Q5JMA6-1 | 0.9390 | 0.7365 | 96.6229 |
| 3 | 6Q76-1_A0A0P0Y5V7-1 | 0.9004 | 0.7364 | 93.0547 |
| 168 | 6Q76-1_6Q76A-1* | 0.8808 | 0.7250 | 95.7609 |
| 242 | 6Q76-1_Q6EPT2-1 | 0.9138 | 0.7220 | 96.2785 |
| 282 | 6Q76-1_Q8S5W0-1 | 0.8757 | 0.7208 | 93.9732 |
| 292 | 6Q76-1_Q2QSQ7-1 | 0.8921 | 0.7202 | 94.3160 |
| 364 | 6Q76-1_Q6EPT4-1 | 0.9038 | 0.7172 | 94.1533 |
| 370 | 6Q76-1_Q7XJV3-1 | 0.8622 | 0.7171 | 94.1851 |
| 412 | 6Q76-1_Q6YY34-1 | 0.8673 | 0.7156 | 93.7823 |
| 474 | 6Q76-1_Q0J314-1 | 0.8863 | 0.7136 | 94.8359 |
| 497 | 6Q76-1_A0A0P0VKX7-1 | 0.8541 | 0.7129 | 91.9085 |
| 498 | 6Q76-1_A0A0N7KFK3-1 | 0.8571 | 0.7128 | 93.3532 |
| 502 | 6Q76-1_Q6YY33-1 | 0.8609 | 0.7127 | 92.9624 |
| 729 | 6Q76-1_Q0JCK8-1 | 0.8183 | 0.7016 | 91.1076 |
| 749 | 6Q76-1_Q6YY31-1 | 0.8251 | 0.7001 | 89.5552 |
| 753 | 6Q76-1_Q7XJV0-1 | 0.8064 | 0.6999 | 89.1890 |
| 772 | 6Q76-1_A0A0P0WB87-1 | 0.8431 | 0.6985 | 89.4861 |
| 116 | 6Q76-1_E9KPB5-1** | 0.6884 | 0.5947 | 74.9171 |
| **Rank by Interface pLDDT score** | | | | |
| rank | complex | ipTM | pDockQ | interface_pLDDT |
| 1 | 6Q76-1_Q5Z6P5-1 | 0.8949 | 0.7216 | 97.8734 |
| 2 | 6Q76-1_Q655X0-1 | 0.8959 | 0.7276 | 97.5381 |
| 3 | 6Q76-1_Q7XKG5-1 | 0.9109 | 0.7291 | 97.5121 |
| 46 | 6Q76-1_Q6EPT2-1 | 0.9138 | 0.7220 | 96.2785 |
| 65 | 6Q76-1_6Q76A-1* | 0.8808 | 0.7250 | 95.7609 |
| 104 | 6Q76-1_Q0J314-1 | 0.8863 | 0.7136 | 94.8359 |
| 132 | 6Q76-1_Q2QSQ7-1 | 0.8921 | 0.7202 | 94.3160 |
| 143 | 6Q76-1_Q7XJV3-1 | 0.8622 | 0.7171 | 94.1851 |
| 146 | 6Q76-1_Q6EPT4-1 | 0.9038 | 0.7172 | 94.1533 |
| 164 | 6Q76-1_Q8S5W0-1 | 0.8757 | 0.7208 | 93.9732 |
| 173 | 6Q76-1_Q6YY34-1 | 0.8673 | 0.7156 | 93.7823 |
| 201 | 6Q76-1_A0A0N7KFK3-1 | 0.8571 | 0.7128 | 93.3532 |
| 235 | 6Q76-1_Q6YY33-1 | 0.8609 | 0.7127 | 92.9624 |
| 323 | 6Q76-1_A0A0P0VKX7-1 | 0.8541 | 0.7129 | 91.9085 |
| 385 | 6Q76-1_Q0JCK8-1 | 0.8183 | 0.7016 | 91.1076 |
| 506 | 6Q76-1_Q6YY31-1 | 0.8251 | 0.7001 | 89.5552 |
| 510 | 6Q76-1_A0A0P0WB87-1 | 0.8431 | 0.6985 | 89.4861 |
| 529 | 6Q76-1_Q7XJV0-1 | 0.8064 | 0.6999 | 89.1890 |
| 114 | 6Q76-1_E9KPB5-1** | 0.6884 | 0.5947 | 74.9171 |

**Supplementary Table 2.** Ranking of complexes of AVR-Pia with true binder and homologous sequences of the true binder in rice. The top scored complexes overall (not homologs) are shaded grey. 6Q76 is the PDB ID of the experimentally known complex from which the AVR-Pia sequence was obtained. * Complex with true binder trimmed to only the portion in PDB 6Q76 chain A

** Complex with full chain of true binder

| **Rank by ipTM score** | | | | |
| --- | --- | --- | --- | --- |
| rank | complex | ipTM | pDockQ | interface_pLDDT |
| 1 | 6R8K-1_A0A0P0Y5A4-1 | 0.9544 | 0.7326 | 89.2781 |
| 2 | 6R8K-1_A0A0P0WTA7-1 | 0.9367 | 0.7401 | 94.1872 |
| 3 | 6R8K-1_A0A0P0YB11-1 | 0.9165 | 0.7314 | 86.0428 |
| 114 | 6R8K-1_Q7XJV3-1 | 0.8290 | 0.7321 | 91.7040 |
| 211 | 6R8K-1_Q6EPT2-1 | 0.8069 | 0.7239 | 86.8982 |
| 237 | 6R8K-1_6R8KA-1* | 0.8007 | 0.7268 | 90.8395 |
| 240 | 6R8K-1_A0A0N7KFK3-1 | 0.8004 | 0.7330 | 93.2785 |
| 315 | 6R8K-1_Q6YY31-1 | 0.7850 | 0.7303 | 92.2765 |
| 428 | 6R8K-1_Q6EPT4-1 | 0.7668 | 0.7179 | 87.0102 |
| 486 | 6R8K-1_Q0JCK8-1 | 0.7562 | 0.7255 | 91.2638 |
| 3419 | 6R8K-1_D5L9G5-1** | 0.3917 | 0.3387 | 61.3701 |
| **Rank by pDockQ score** | | | | |
| rank | complex | ipTM | pDockQ | interface_pLDDT |
| 1 | 6R8K-1_A0A0P0WTA7-1 | 0.9367 | 0.7401 | 94.1872 |
| 2 | 6R8K-1_G9LZD7-1 | 0.8740 | 0.7393 | 90.3427 |
| 3 | 6R8K-1_Q5NAK8-1 | 0.8294 | 0.7370 | 91.0218 |
| 36 | 6R8K-1_A0A0N7KFK3-1 | 0.8004 | 0.7330 | 93.2785 |
| 45 | 6R8K-1_Q7XJV3-1 | 0.8290 | 0.7321 | 91.7040 |
| 72 | 6R8K-1_Q6YY31-1 | 0.7850 | 0.7303 | 92.2765 |
| 140 | 6R8K-1_6R8KA-1* | 0.8007 | 0.7268 | 90.8395 |
| 174 | 6R8K-1_Q0JCK8-1 | 0.7562 | 0.7255 | 91.2638 |
| 197 | 6R8K-1_Q6EPT2-1 | 0.8069 | 0.7239 | 86.8982 |
| 272 | 6R8K-1_Q6EPT4-1 | 0.7668 | 0.7179 | 87.0102 |
| 523 | 6R8K-1_D5L9G5-1** | 0.3917 | 0.3387 | 61.3701 |
| **Rank by Interface pLDDT score** | | | | |
| rank | complex | ipTM | pDockQ | interface_pLDDT |
| 1 | 6R8K-1_Q9SDG5-1 | 0.8060 | 0.5720 | 96.7147 |
| 2 | 6R8K-1_B7F9Y7-1 | 0.7806 | 0.7282 | 96.4087 |
| 3 | 6R8K-1_Q0J845-1 | 0.8464 | 0.7160 | 96.3657 |
| 59 | 6R8K-1_A0A0N7KFK3-1 | 0.8004 | 0.7330 | 93.2785 |
| 87 | 6R8K-1_Q6YY31-1 | 0.7850 | 0.7303 | 92.2765 |
| 104 | 6R8K-1_Q7XJV3-1 | 0.8290 | 0.7321 | 91.7040 |
| 118 | 6R8K-1_Q0JCK8-1 | 0.7562 | 0.7255 | 91.2638 |
| 130 | 6R8K-1_6R8KA-1* | 0.8007 | 0.7268 | 90.8395 |
| 263 | 6R8K-1_Q6EPT4-1 | 0.7668 | 0.7179 | 87.0102 |
| 264 | 6R8K-1_Q6EPT2-1 | 0.8069 | 0.7239 | 86.8982 |
| 520 | 6R8K-1_D5L9G5-1** | 0.3917 | 0.3387 | 61.3701 |

**Supplementary Table 3.** Ranking of complexes of AVR-Pik with true binder and homologous sequences of the true binder in rice. The top scored complexes overall (not homologs) are shaded grey. 6R8K is the PDB ID of the experimentally known complex from which the AVR-Pik sequence was obtained. * Complex with true binder trimmed to only the portion in PDB 6R8K chain A

** Complex with full chain of true binder

**Supplementary Figure 1.** Violin plots of the ipTM, pDockQ, and ipLDDT scores of the top clusters for the three case studies. The width of each curve corresponds with the approximate frequency of data points in each region. The clusters containing the true binding topologies are encircled.

**Supplementary Figure 2.** ipTM scores of all the complexes for each proteome-wide screen with an ipTM score > 0.5. The complexes in the top clusters are shown as colored diamonds, and the complexes that are not in one of the top clusters are gray diamonds. The clusters with the true binders are highlighted with dotted lines.

**Supplementary Figure 3.** Resulting clusters after running AlphaCRV on the dimeric complexes of UspA, TRCF, and LPTE against the *E. coli* proteome. UspA is known to form homodimers, and AlphaCRV placed the homodimeric complex it in a cluster of size 5 (highlighted in yellow), which was enough to recognize it as a promising binding topology in comparison to the other clusters. The sequences of the true binders of TRCF and LPTE lack homologues in the *E. coli* proteome, so even though AlphaFold predicted the true complexes with high quality (ipTM > 0.75), they were not part of any clusters. Center top: largest cluster identified for uspA, which contains the true homodimeric complex. Center middle and bottom: AlphaFold predicted complexes (blue cartoons) superimposed to the PDB structures of the true complexes for TRCF and LPTE (grey cartoons, PDB IDs 4dfc top and 4rhb middle). Right: AlphaFold predictions of the true complexes colored by pLDDT score. Dark blue regions are modeled with high confidence (pLDDT > 90), light blue with high confidence (pLDDT > 70), yellow with low confidence (pLDDT > 50), and red regions have very low confidence (pLDDT < 50).


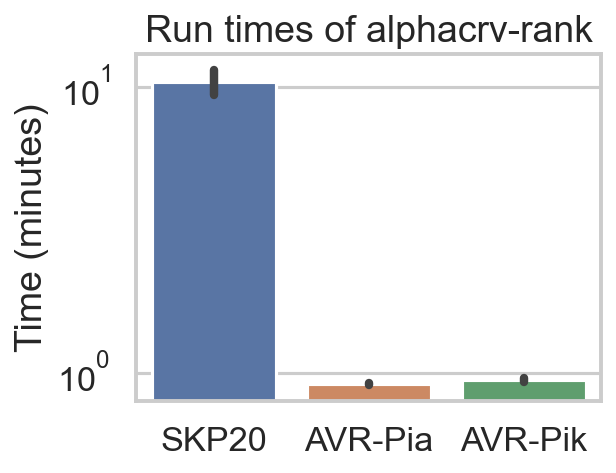

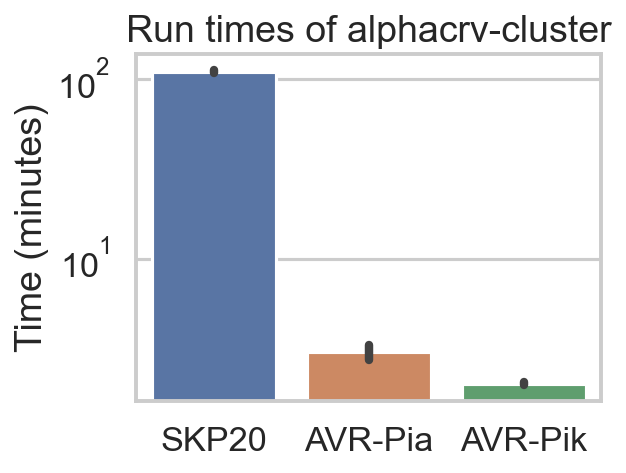

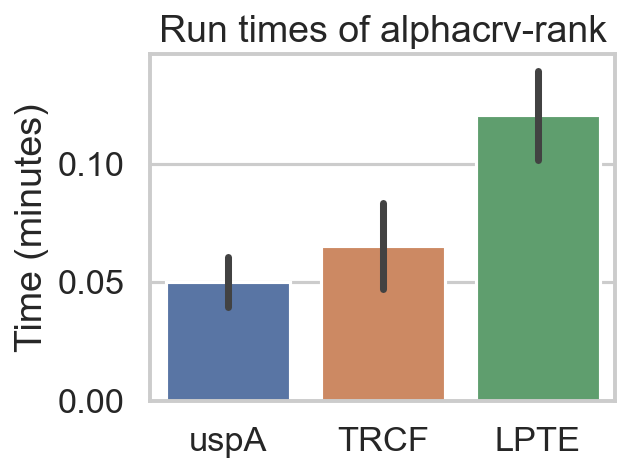

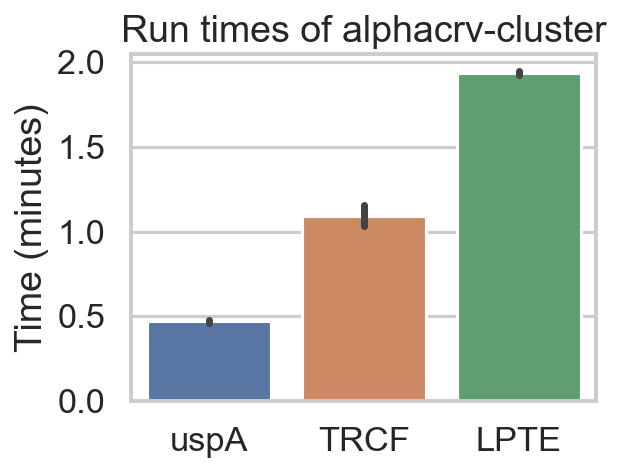


**Supplementary Figure 4.** Run times of AlphaCRV in a machine using 8 CPU cores for the test cases on the rice proteome (top) and the *E. coli* proteome (bottom). The bars represent the mean run time from 3 independent runs in each case, and the lines in the middle show the standard deviation. The run time of the alphacrv-cluster command increases to the square of the number of complexes in the largest cluster. The number of complexes processed was 712, 99, 65, 28, 48, and 68 for SKP20, AVR-Pia, AVR-Pik, uspA, TRCF, and LPTE, respectively.
