# Supplementary material for: AlphaCRV: a pipeline for identifying accurate binder topologies in mass-modeling with AlphaFold
Source: Bioinform Adv. 2024 Sep 6;4(1):vbae131. doi: 10.1093/bioadv/vbae131 (PMC11405088; doi:10.1093/bioadv/vbae131)
Supplement: vbae131_Supplementary_Data [file vbae131_supplementary_data.zip › vbae131_Supplementary_Data/Supplementary_Note_2024-08.pdf]

Clustering SKP1

This notebook will guide you through the process of clustering 712 complexes of the SKP1 protein against a sample of rice proteins. All of the models are dimeric. The 712 models originate from a proteome-wide screen of SKP1 against the rice proteome (O. sativa subsp. japonica, 43,000+ initial models). To select this sample we first ran AlphaCRV on all the models, and the 712 structures are part of some of the best clusters that were identified. These are many more than the other examples because the largest cluster in this case has around 700 structures, which might be due to the higher number of homologues of SKP1 compared to the two AVR proteins in the other examples. Running the pipeline on these models should be enough to reproduce the results and give you an idea of the workflow.

Prerequisites

- Install AlphaCRV on a conda environment and activate it
- You can download the sequences, resulting clusters, and PyMOL sessions for this example at Zenodo
- The models for this example are very heavy (>140 GB), so we will provide them upon request if you wish to run AlphaCRV on them. Please contact us at stefan.arold@kaust.edu.sa, or francisco.guzmanvega@kaust.edu.sa

1. Cluster the models with the `clustering` command

Run the following command to cluster the models. Make sure to change the paths according to your system:

```
alphacrv-cluster \
  --bait ./examples/SKP1/SKP1.fasta \
  --binders ./examples/SKP1/SKP1_binders.fasta \
  --models_dir ./examples/SKP1/SKP1_vs_rice_models \
  --destination ./examples/SKP1/SKP1_vs_rice_clusters \
  --cpus 8
```

After collecting the quality scores from the models in `--models_dir`, it will count how many models are there with an ipTM score higher than the threshold (0.75 by default). It will prompt you to confirm or to modify the threshold. After that, it will proceed with the clustering.

This run will take considerably longer than the two other examples, because of the large size of the first cluster. The full output is as follows:

```
(env) AlphaCRV$ alphacrv-cluster \
--bait ./examples/SKP1/SKP1.fasta \
--binders ./examples/SKP1/SKP1_binders.fasta \
--models_dir ./examples/SKP1/SKP1_vs_rice_models \
--destination ./examples/SKP1/SKP1_vs_rice_clusters \
--cpus 8
INFO:root:Getting quality scores for models in examples/SKP1/
SKP1_vs_rice_models...
INFO:root:Found 712 model directories with quality scores.
Will select 712 models with iptm >= 0.75. Press enter to
continue, or enter a new threshold:
INFO:root:Trimming binder molecules to keep only regions with
an average PAE against the bait of up to 10.0...
INFO:root:Processed 712 complexes.
INFO:root:Writing trimmed sequences to fasta file.
INFO:root:Running sequence clustering...
INFO:root:Processing output...
INFO:root:Running structural clustering...
INFO:root:Processing output...
INFO:root:NOT ALL REPRESENTATIVES COME FROM CHAIN B
INFO:root:Aligning all vs all members of each cluster...
INFO:root:Aligning cluster 1 of 4...
INFO:root:695 members.
INFO:root:Aligning cluster 2 of 4...
INFO:root:6 members.
INFO:root:Aligning cluster 3 of 4...
INFO:root:2 members.
INFO:root:Aligning cluster 4 of 4...
INFO:root:9 members.
INFO:root:Calculating median alignment scores...
INFO:root:Done!!
```

After this step you will have a directory with the following structure:

```
(env) AlphaCRV$ ll examples/SKP1/SKP1_vs_rice_clusters/
total 248K
-rw-r--r-- 1 example g-example  15K Jan  4 13:39
binders_regions.csv
drwxr-xr-x 1 example g-example    0 Jan  4 15:18
merged_clusters/
```

```
drwxr-xr-x 1 example g-example    0 Jan  4 13:39
pdbs_trimmed/
drwxr-xr-x 1 example g-example    0 Jan  4 13:39 seqclusters/
drwxr-xr-x 1 example g-example    0 Jan  4 13:39 strclusters/
-rw-r--r-- 1 example g-example 230K Jan  4 13:39
trimmed_binders.fasta
```

Now let's look at some of the important files:

In [ ]:
```
from pathlib import Path
import pandas as pd
```

In [ ]:
```
results_dir = Path('./SKP1/SKP1_vs_rice_clusters/')
```

See clusters

The `merged_clusters.csv` file contains the list of models with their corresponding sequence, structure and merged clusters. It also has the quality scores provided by AlphaFold.

In [ ]:
```
clusters = pd.read_csv(results_dir / 'merged_clusters/merged_clusters.csv')
```

In [ ]:
```
clusters
```

Out[ ]:

	complex	str_rep	seq_rep	merged_rep	
**0**	8IF6-1_Q6Z1A9-1	A0A0P0Y2U6.pdb_B	A0A0N7KPE6	A0A0P0XHF4.pdb_B	
**1**	8IF6-1_Q7XSL8-1	A0A0P0WQD9.pdb_B	A0A0P0VJA5	A0A0P0XHF4.pdb_B	
**2**	8IF6-1_Q5Z8K3-1	Q6ZDH1.pdb_B	Q5Z8K3	A0A0P0XHF4.pdb_B	
**3**	8IF6-1_Q5VMP0-1	A0A0P0WQD9.pdb_B	Q5VMP0	A0A0P0XHF4.pdb_B	
**4**	8IF6-1_A0A0P0WG98-1	Q5Z7U2.pdb_B	C7J8M0	A0A0P0XHF4.pdb_B	A(
**...**	...	...	...	...	
**707**	8IF6-1_Q0JK63-1	Q0JK63.pdb_B	Q0JK63	A0A0P0XHF4.pdb_B	
**708**	8IF6-1_Q53WL8-1	Q6ZDH1.pdb_B	A0A0P0WPS2	A0A0P0XHF4.pdb_B	
**709**	8IF6-1_A3AQW3-1	A0A0N7KT42.pdb_B	A3AQW3	A0A0P0XHF4.pdb_B	
**710**	8IF6-1_Q6ZKB8-1	Q0DQG8.pdb_B	Q6ZKB8	A0A0P0XHF4.pdb_B	
**711**	8IF6-1_Q0DEJ9-1	A0A0P0XE77.pdb_B	A0A0P0WSK5	A0A0P0XHF4.pdb_B	

712 rows × 7 columns

The columns of the `clusters` DataFrame are:

- `complex` : The name of the complex. This is the same name as the directory where

the model is stored.

- `str_rep` : Name of the structure cluster representative
- `seq_rep` : Name of the sequence cluster representative
- `merged_rep` : Name of the merged cluster representative (sequence + structure)
- `member` : The ID of the binder protein
- `iptm` : The ipTM score of the model
- `iptm+ptm` : The ipTM+PTM score of the model (it is calculated by AlphaFold as 0.8*ipTM + 0.2*pTM)

See the number of different merged clusters:

In [ ]:
```
clusters.merged_rep.unique().shape
```

Out[ ]:
```
(4,)
```

For this example, the models and sequences from the 712 binder proteins were summarized in 4 clusters. Much fewer structures to sort through!

See alignment scores

Alignment scores are calcualted for each cluster by aligning all vs all members of the cluster.

In [ ]:
```
alignment_scores = pd.read_csv(results_dir / 'merged_clusters/alignment_scor
```

In [ ]:
```
alignment_scores.head()
```

Out[ ]:

	cluster	ref	member	tmscore_ref	tmscore_m	aligned_lengtl
0	A0A0P0XHF4.pdb_B	Q6Z1A9	Q7XSL8	0.40236	0.25976	21!
1	A0A0P0XHF4.pdb_B	Q6Z1A9	Q5Z8K3	0.38146	0.28447	18!
2	A0A0P0XHF4.pdb_B	Q6Z1A9	Q5VMP0	0.42881	0.23565	41!
3	A0A0P0XHF4.pdb_B	Q6Z1A9	A0A0P0WG98	0.38724	0.35182	23!
4	A0A0P0XHF4.pdb_B	Q6Z1A9	Q67UX0	0.40441	0.30453	26:

The columns of the `alignment_scores` DataFrame are:

- `cluster` : The name of the cluster
- `ref` : Binder ID of the reference structure in the alignment
- `member` : Binder ID of the second structure in the alignment
- `tmscore_ref` : TM-score based on the reference structure
- `tmscore_m` : TM-score based on the second structure
- `aligned_length` : Length of the alignment
- `rmsd` : RMSD of the alignment

Based on these scores, the median scores are calculated for each cluster member to find the best representative of the cluster (the one with lowest RMSD score to the other members).

Read median scores and find top clusters

Now we can rank the clusters based on the median alignment scores of the cluster representatives:

In [ ]:
```
median_scores = pd.read_csv(results_dir / 'merged_clusters/median_scores.csv
```

In [ ]:
```
median_scores.shape
```

Out[ ]:
```
(712, 7)
```

The `median_scores` DataFrame contains the median alignment scores of each cluster member when aligned to all other members of the same cluster.

In [ ]:
```
median_scores.head()
```

Out[ ]:

	cluster	member	tmscore	rmsd	aligned_length	cluster_size	frac
**0**	A0A0P0VR14.pdb_B	A0A0P0VR14	0.32871	18.64	208.0	6.0	
**1**	A0A0P0VR14.pdb_B	Q2R448	0.85991	6.04	445.0	6.0	
**2**	A0A0P0VR14.pdb_B	Q6K6K8	0.85162	4.42	445.0	6.0	
**3**	A0A0P0VR14.pdb_B	Q7XKU0	0.49110	17.19	405.0	6.0	
**4**	A0A0P0VR14.pdb_B	Q7XL60	0.82004	4.95	455.0	6.0	

The columns of the `median_scores` DataFrame are:

- `cluster` : The name of the cluster
- `member` : ID of the cluster member (binder molecule)
- `tmscore` : Median TM-score of the complex against all other complexes in this cluster
- `rmsd` : Median RMSD of the complex against all other complexes in this cluster
- `aligned_length` : Median length of the alignment
- `cluster_size`
- `fraction_binder` : In average, how much of the binder molecule is included in the alignments of this complex against all other complexes (calculated as `(aligned_length - bait_length)/binder_length`). This is just meant to be an approximation of how complete the aligmnents are for this cluster member.

The next step is to select the cluster representatives. For this, we first need to filter out the cluster members with poor quality alignments, according to the following criteria:

- Small size
- Low median TM-score
- High median RMSD
- Low fraction of the binder aligned in the cluster representative

In [ ]:
```
# Select the clusters with the following criteria:
select = ((median_scores.cluster_size >= 5) & \
            (median_scores.tmscore >= 0.2) & \
            (median_scores.fraction_binder >= 0.2) & \
            (median_scores.rmsd <= 15))
median_scores_filtered = median_scores[select]
```

In [ ]:
```
median_scores_filtered.shape
```

Out[ ]:
```
(169, 7)
```

See how many clusters are left after filtering:

In [ ]:
```
median_scores_filtered.cluster.unique().shape
```

Out[ ]:
```
(3,)
```

Function to format tables:

In [ ]:
```
import seaborn as sns
cm_r = sns.color_palette("mako_r", as_cmap=True)
cm = sns.color_palette("mako", as_cmap=True)
```

In [ ]:
```
def make_pretty(styler):
    styler.format(precision=2)
    styler.background_gradient(axis=0, cmap=cm_r, subset=pd.IndexSlice[:,"cl
    styler.background_gradient(axis=0, cmap=cm_r, subset=pd.IndexSlice[:,"tm
    styler.background_gradient(axis=0, cmap=cm, subset=pd.IndexSlice[:,"rmsd
    styler.background_gradient(axis=0, cmap=cm_r, subset=pd.IndexSlice[:,"fr
    return styler
```

RESULT 1: See clusters ranked by RMSD

Finally, we can rank the clusters and see which ones have a good combination of low RMSD and large cluster size. These ones are the most likely to contain the true binder.

In [ ]:
```
# Select the rows with the minimum RMSD for each cluster
select = median_scores_filtered.groupby('cluster').rmsd.idxmin()
columns = ['cluster', 'tmscore', 'rmsd', 'cluster_size', 'fraction_binder']
median_scores_filtered.loc[select,columns].sort_values(by='rmsd').style.pipe
```

Out[ ]:

	cluster	tmscore	rmsd	cluster_size	fraction_binder
**19**	A0A0P0XHF4.pdb_B	0.94	1.53	695.00	1.00
**2**	A0A0P0VR14.pdb_B	0.85	4.42	6.00	0.90
**704**	Q65XD1.pdb_B	0.70	13.05	9.00	0.93

Here we can see that the cluster `B7ESQ3.pdb_B` has the lowest median RMSD and the highest median TM-score. It also has a very large size with 695 members!

RESULT 2: See clusters ranked by size

In [ ]:

```
# Select the rows with the minimum RMSD for each cluster
select = median_scores_filtered.groupby('cluster').rmsd.idxmin()
columns = ['cluster', 'tmscore', 'rmsd', 'cluster_size', 'fraction_binder']
median_scores_filtered.loc[select, columns].sort_values(by='cluster_size', a
```

Out[ ]:

	cluster	tmscore	rmsd	cluster_size	fraction_binder
**19**	A0A0P0XHF4.pdb_B	0.94	1.53	695.00	1.00
**704**	Q65XD1.pdb_B	0.70	13.05	9.00	0.93
**2**	A0A0P0VR14.pdb_B	0.85	4.42	6.00	0.90

`B7ESQ3.pdb_B` is also the largest cluster. So we managed to reduce more than 43,000 starting models to only one excellent cluster!

For this example we know that in our list of candidate binders there are 5 homologues of the true binder protein. We can find out which clusters contain these homologues:

In [ ]:

```
homologues = ['Q5VMP0', 'A0A0N7KEW0', 'Q8RZQ3', 'Q69X07', 'A0A0P0Y6A8']
```

In [ ]:

```
clusters[clusters.member.isin(homologues)][['complex','merged_rep','iptm','i
```

Out[ ]:

	complex	merged_rep	iptm	iptm+ptm
**3**	8IF6-1_Q5VMP0-1	A0A0P0XHF4.pdb_B	0.899220	0.894956
**28**	8IF6-1_A0A0P0Y6A8-1	A0A0P0XHF4.pdb_B	0.893004	0.886242
**321**	8IF6-1_Q8RZQ3-1	A0A0P0XHF4.pdb_B	0.867056	0.855604
**372**	8IF6-1_A0A0N7KEW0-1	A0A0P0XHF4.pdb_B	0.862492	0.844927
**516**	8IF6-1_Q69X07-1	A0A0P0XHF4.pdb_B	0.847566	0.808583

They are all in the top cluster!

2. Make pymol sessions for the top clusters

with `make_pymol_sessions`

Run the following command to select the top clusters that we saw above, make pymol sessions of the top clusters, and optionally do structural clustering on each cluster to find subclusters:

```
alphacrv-rank \
  --clusters_dir ./examples/AVRPia/AVRPia_vs_rice_clusters \
  --min_members 5 \
  --min_tmscore 0.2 \
  --max_rmsd 15 \
  --cluster_clusters
```

The program will show you the top clusters that will be used to make the pymol sessions. You can press `Enter` to continue, or exit the program with `Ctrl+C` to change the filtering parameters.

```
(env) AlphaCRV$ alphacrv-rank \
--clusters_dir ./examples/SKP1/SKP1_vs_rice_clusters \
--min_members 5 \
--min_tmscore 0.2 \
--max_rmsd 15 \
--cluster_clusters
INFO:root:Identified 3 top clusters.
INFO:root:Top clusters:

            cluster   tmscore    rmsd  cluster_size
fraction_binder
0  A0A0P0XHF4.pdb_B  0.941210   1.530         695.0
1.00000
1      Q65XD1.pdb_B  0.704515  13.055           9.0
0.93000
2  A0A0P0VR14.pdb_B  0.851620   4.420           6.0
0.90301

Press Enter to continue, or Ctrl+C to exit and select
different filtering parameters:
INFO:root:Copying pdbs from the top clusters...
INFO:root:Making Pymol sessions...
PyMOL>select chain B AND model A0A0P0VR14_repB
 Selector: selection "sele" defined with 4621 atoms.
PyMOL>bg white
PyMOL>set ray_shadow, 0
 Setting: ray_shadow set to off.
PyMOL>color grey80
 Executive: Colored 42707 atoms.
PyMOL>select chain A
 Selector: selection "sele" defined with 16020 atoms.
PyMOL>color slate, sele
 Executive: Colored 16020 atoms.
```

```
PyMOL>delete all
PyMOL>select chain B AND model A0A0P0XHF4_repB
 Selector: selection "sele" defined with 646 atoms.
PyMOL>bg white
PyMOL>set ray_shadow, 0
 Setting: ray_shadow set to off.
PyMOL>color grey80
 Executive: Colored 5270014 atoms.
PyMOL>select chain A
 Selector: selection "sele" defined with 1844949 atoms.
PyMOL>color slate, sele
 Executive: Colored 1844949 atoms.
PyMOL>delete all
PyMOL>select chain B AND model Q65XD1_repB
 Selector: selection "sele" defined with 3154 atoms.
PyMOL>bg white
PyMOL>set ray_shadow, 0
 Setting: ray_shadow set to off.
PyMOL>color grey80
 Executive: Colored 76932 atoms.
PyMOL>select chain A
 Selector: selection "sele" defined with 24030 atoms.
PyMOL>color slate, sele
 Executive: Colored 24030 atoms.
PyMOL>delete all
INFO:root:Clustering clusters...
INFO:root:Clustering A0A0P0VR14.pdb_B
INFO:root:Running structural clustering...
INFO:root:Processing output...
INFO:root:Clustering A0A0P0XHF4.pdb_B
INFO:root:Running structural clustering...
INFO:root:Processing output...
INFO:root:Clustering Q65XD1.pdb_B
INFO:root:Running structural clustering...
INFO:root:Processing output...
INFO:root:Done!!
```

This command should create the following files in the `./examples/SKP1/SKP1_vs_rice_clusters/merged_clusters/` / `merged_clusters` directory:

- `clustered_clusters.csv` : Contains the subclusters for each of the top clusters.
- `cluster_<cluster_ID>/` : Contains the PDBs of each cluster, and a PyMol session with the cluster members.
- `cluster_<cluster_ID>_clusters/` : Contains the results of the `foldseek easy-cluster` run on the cluster members.

Read clustered clusters

The following DataFrame contains the subclusters for each of the top clusters:

In [ ]:
```
clustered_clusters = pd.read_csv(results_dir / 'merged_clusters/clustered_cl
```

In [ ]:
```
clustered_clusters.head()
```

Out[ ]:

	subcluster_rep	member	cluster
0	A0A0P0VR14.pdb_B	A0A0P0VR14	A0A0P0VR14.pdb_B
1	A0A0P0VR14.pdb_B	Q7XKU0	A0A0P0VR14.pdb_B
2	A0A0P0VR14.pdb_B	Q7XL60	A0A0P0VR14.pdb_B
3	A0A0P0VR14.pdb_B	Q6K6K8	A0A0P0VR14.pdb_B
4	A0A0P0VR14.pdb_B	Q2R448	A0A0P0VR14.pdb_B

In [ ]:
```
clustered_clusters.subcluster_rep.value_counts().head()
```

Out[ ]:
```
subcluster_rep
Q6ZDH1.pdb_B        223
A0A0P0WQD9.pdb_B    116
A0A0P0Y2U6.pdb_B     51
Q5Z7U2.pdb_B         35
Q0DQG8.pdb_B         34
Name: count, dtype: int64
```

In [ ]:
```
median_scores_subclusters = pd.merge(median_scores, clustered_clusters, on='
# Add subcluster size
sizes = clustered_clusters.subcluster_rep.value_counts()
median_scores_subclusters['subcluster_size'] = median_scores_subclusters.sub
median_scores_subclusters.rename(columns={'subcluster_rep':'subcluster'}, in
```

In [ ]:
```
median_scores_subclusters.head()
```

Out[ ]:

	cluster_x	member	tmscore	rmsd	aligned_length	cluster_size	frac
0	A0A0P0VR14.pdb_B	A0A0P0VR14	0.32871	18.64	208.0	6.0	
1	A0A0P0VR14.pdb_B	Q2R448	0.85991	6.04	445.0	6.0	
2	A0A0P0VR14.pdb_B	Q6K6K8	0.85162	4.42	445.0	6.0	
3	A0A0P0VR14.pdb_B	Q7XKU0	0.49110	17.19	405.0	6.0	
4	A0A0P0VR14.pdb_B	Q7XL60	0.82004	4.95	455.0	6.0	

See subclusters ranked by size:

In [ ]:
```
def make_pretty(styler):
    styler.format(precision=2)
    styler.background_gradient(axis=0, cmap=cm_r, subset=pd.IndexSlice[:,"su
    styler.background_gradient(axis=0, cmap=cm_r, subset=pd.IndexSlice[:,"tm
    styler.background_gradient(axis=0, cmap=cm, subset=pd.IndexSlice[:,"rmsd
```

```
    styler.background_gradient(axis=0, cmap=cm_r, subset=pd.IndexSlice[:,"fr
    return styler
```

In [ ]:

```
# Select the rows with the minimum RMSD for each cluster
select = median_scores_subclusters.groupby('subcluster').rmsd.idxmin()
columns = ['subcluster', 'tmscore', 'rmsd', 'subcluster_size', 'fraction_bin
median_scores_subclusters.loc[select, columns].sort_values(by='subcluster_si
                                                 ascending=False).style.pipe(make
```

Out[ ]:

	subcluster	tmscore	rmsd	subcluster_size	fraction_binder
**213**	Q6ZDH1.pdb_B	0.30	7.94	223.00	0.07
**367**	A0A0P0WQD9.pdb_B	0.29	8.05	116.00	0.10
**69**	A0A0P0Y2U6.pdb_B	0.38	8.20	51.00	0.16
**149**	Q5Z7U2.pdb_B	0.42	7.34	35.00	0.16
**691**	Q0DQG8.pdb_B	0.30	8.86	34.00	0.05
**533**	Q75J50.pdb_B	0.45	8.55	27.00	0.21
**374**	Q0JPJ6.pdb_B	0.35	9.29	19.00	0.05
**480**	A0A0P0XHI5.pdb_B	0.92	1.85	14.00	0.95
**310**	A0A0P0X3V6.pdb_B	0.60	7.79	14.00	0.28
**57**	A0A0P0VHP5.pdb_B	0.91	2.46	12.00	0.96
**126**	Q7XAK4.pdb_B	0.91	2.27	9.00	0.94
**520**	Q7EY32.pdb_B	0.94	1.56	9.00	0.97
**49**	A0A0P0V3R7.pdb_B	0.34	10.34	9.00	0.07
**469**	Q8LJA9.pdb_B	0.31	10.07	8.00	0.06
**707**	Q65XD1.pdb_B	0.46	17.29	8.00	0.92
**65**	A0A0P0XE00.pdb_B	0.71	7.98	7.00	0.80
**418**	A0A0P0X458.pdb_B	0.93	1.76	7.00	0.98
**245**	Q0JB10.pdb_B	0.73	8.09	6.00	0.87
**16**	Q5N762.pdb_B	0.79	6.56	6.00	0.79
**203**	A0A0P0W419.pdb_B	0.55	9.55	6.00	0.26
**2**	A0A0P0VR14.pdb_B	0.85	4.42	6.00	0.90
**81**	A0A0P0VRN2.pdb_B	0.60	7.75	5.00	0.36
**362**	Q0JK63.pdb_B	0.27	9.57	5.00	0.04
**88**	A0A0P0XEW7.pdb_B	0.93	1.84	5.00	0.98
**656**	A0A0P0VNI4.pdb_B	0.82	5.88	5.00	0.83
**259**	Q7FAH1.pdb_B	0.61	8.93	5.00	0.31
**689**	Q75IQ5.pdb_B	0.89	2.19	4.00	0.98
**171**	A0A0P0Y5S9.pdb_B	0.69	7.33	4.00	0.53
**650**	A0A0N7KD81.pdb_A	0.33	6.99	4.00	0.04
**394**	A0A0P0VNF2.pdb_B	0.92	1.83	4.00	0.98
**318**	A0A0P0XE77.pdb_B	0.91	2.16	4.00	0.95
**145**	A0A0P0XNQ3.pdb_B	0.92	1.78	3.00	0.93

	subcluster	tmscore	rmsd	subcluster_size	fraction_binder
**37**	A0A0N7KT42.pdb_B	0.94	1.63	3.00	0.97
**188**	A0A0P0XFT5.pdb_B	0.49	10.24	3.00	0.13
**373**	Q109D8.pdb_B	0.60	8.80	2.00	0.33
**588**	A0A0P0XHF4.pdb_B	0.93	1.99	2.00	1.00
**693**	A0A0P0X4D4.pdb_B	0.92	1.86	2.00	0.97
**173**	A0A0P0XCY3.pdb_B	0.74	7.05	2.00	0.79
**60**	A0A0P0VGE5.pdb_B	0.86	2.88	2.00	0.93
**369**	B7ESQ3.pdb_B	0.91	2.32	2.00	0.94
**270**	A2ZPC4.pdb_B	0.32	10.02	2.00	0.06
**77**	A0A0N7KN01.pdb_B	0.51	12.01	2.00	0.26
**508**	Q69J07.pdb_B	0.92	2.21	1.00	0.98
**62**	A0A0P0VHD9.pdb_B	0.91	2.03	1.00	0.95
**45**	A0A0P0V091.pdb_B	0.42	11.86	1.00	0.13
**517**	Q69X07.pdb_B	0.69	9.89	1.00	0.65
**704**	A0A0P0Y2H6.pdb_B	0.70	13.05	1.00	0.93
**85**	A0A0P0VZH9.pdb_B	0.65	9.68	1.00	0.67
**114**	A0A0P0WDY8.pdb_B	0.81	6.84	1.00	0.87
**401**	Q2QQH8.pdb_B	0.94	1.60	1.00	0.98
**19**	A0A0N7KK85.pdb_B	0.94	1.53	1.00	1.00
**300**	B9G6M6.pdb_B	0.87	2.68	1.00	0.91
**263**	A0A0P0YC15.pdb_B	0.31	12.09	1.00	0.06
**254**	A0A0P0YAA2.pdb_B	0.91	1.94	1.00	0.95
**253**	A0A0P0YA59.pdb_B	0.89	2.38	1.00	0.89
**223**	A0A0P0XSL8.pdb_B	0.89	2.28	1.00	0.87

Now we can look at the most interesting clusters and their subclusters in more detail:

RESULT 1: Cluster A0A0P0XHF4.pdb_B (contains true binder homologs, top cluster by size)

In [ ]:
```
cluster = 'A0A0P0XHF4.pdb_B'
```

See subclusters:

In [ ]:
```
clustered_clusters[clustered_clusters.cluster==cluster].head()
```

Out[ ]:

	subcluster_rep	member	cluster
**6**	A0A0P0VGE5.pdb_B	A0A0P0VGE5	A0A0P0XHF4.pdb_B
**7**	A0A0P0VGE5.pdb_B	Q67W96	A0A0P0XHF4.pdb_B
**8**	A0A0P0VHD9.pdb_B	A0A0P0VHD9	A0A0P0XHF4.pdb_B
**9**	A0A0P0VHP5.pdb_B	A0A0P0VHP5	A0A0P0XHF4.pdb_B
**10**	A0A0P0VHP5.pdb_B	A0A0P0UZC1	A0A0P0XHF4.pdb_B

Count how many subclusters:

In [ ]:
```
clustered_clusters[clustered_clusters.cluster==cluster].subcluster_rep.uniqu
```

Out[ ]:
```
(53,)
```

See the amount of strucures in each subcluster:

In [ ]:
```
(clustered_clusters[clustered_clusters.cluster==cluster].groupby('subcluster
 .size().sort_values(ascending=False).head(15))
```

Out[ ]:
```
subcluster_rep
Q6ZDH1.pdb_B         223
A0A0P0WQD9.pdb_B     116
A0A0P0Y2U6.pdb_B      51
Q5Z7U2.pdb_B          35
Q0DQG8.pdb_B          34
Q75J50.pdb_B          27
Q0JPJ6.pdb_B          19
A0A0P0XHI5.pdb_B      14
A0A0P0X3V6.pdb_B      14
A0A0P0VHP5.pdb_B      12
Q7XAK4.pdb_B           9
Q7EY32.pdb_B           9
A0A0P0V3R7.pdb_B       9
Q8LJA9.pdb_B           8
A0A0P0X458.pdb_B       7
dtype: int64
```

Here we have 53 subclusters!! This cluster has in total 695 strucures, which is a bit too much to load into PyMOL at once. We have to change our strategy. One way to decide if the subclusters are similar or different to each other would be to only look at the subcluster representatives and classify them according to their topology. We will try now three different approaches to visualize this cluster:

Take all members of this cluster together

We can just look at the structures with lowest median RMSD to have an idea of how this

cluster looks:

In [ ]:
```
median_scores[median_scores.cluster==cluster].sort_values(by='rmsd').head()
```

Out[ ]:

	cluster	member	tmscore	rmsd	aligned_length	cluster_size
**19**	A0A0P0XHF4.pdb_B	A0A0N7KK85	0.941210	1.530	210.0	695.0
**520**	A0A0P0XHF4.pdb_B	Q6ASY4	0.938210	1.560	213.0	695.0
**401**	A0A0P0XHF4.pdb_B	Q2QQH8	0.938700	1.600	215.0	695.0
**37**	A0A0P0XHF4.pdb_B	A0A0N7KT42	0.935435	1.630	208.0	695.0
**256**	A0A0P0XHF4.pdb_B	A0A0P0YAQ4	0.935185	1.645	217.0	695.0

We can print out the names of the top 20 models to open them in PyMOL (through the terminal or a command inside of PyMOL)

In [ ]:
```
path_cluster = Path.cwd().resolve()
```

In [ ]:
```
path_cluster = '/Volumes/weka_user/guzmanfj/py/AlphaCRV/examples/'
```

In [ ]:
```
# Print paths to open models in pymol
membs = median_scores[median_scores.cluster==cluster].sort_values(by='rmsd')
' '.join([(path_cluster + str(results_dir) + f'/merged_clusters/cluster_{clu
```

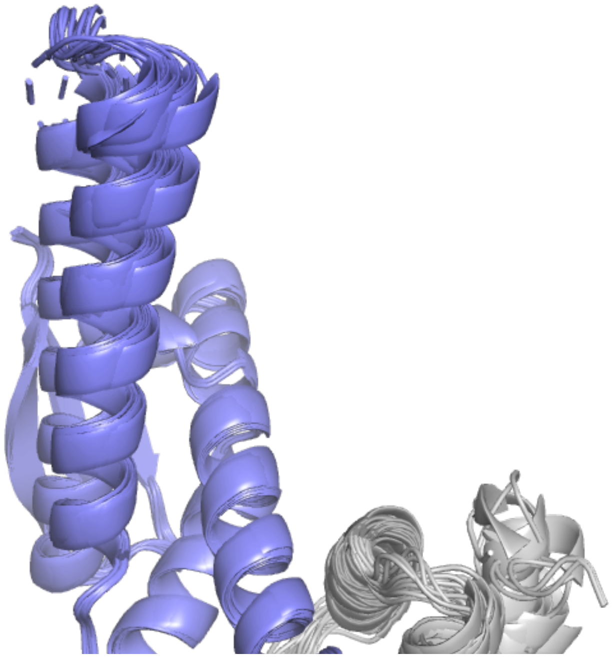

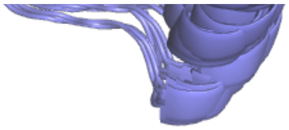

Look at all the subcluster representatives in PyMOL and group them by topology.

In [ ]:
```
subclusters = (clustered_clusters[clustered_clusters.cluster==cluster])
subclusters_ids = subclusters['subcluster_rep'].str.split('.pdb').str[0]
```

See the names of the subclusters:

In [ ]:
```
subclusters.subcluster_rep.unique()
```

Out[ ]:
```
array(['A0A0P0VGE5.pdb_B', 'A0A0P0VHD9.pdb_B', 'A0A0P0VHP5.pdb_B',
       'A0A0P0VNF2.pdb_B', 'A0A0P0VNI4.pdb_B', 'A0A0P0VRN2.pdb_B',
       'A0A0P0VZH9.pdb_B', 'A0A0P0W419.pdb_B', 'A0A0P0X4D4.pdb_B',
       'A0A0P0XCY3.pdb_B', 'A0A0P0XE00.pdb_B', 'A0A0P0XE77.pdb_B',
       'A0A0P0XEW7.pdb_B', 'A0A0P0XFT5.pdb_B', 'A0A0P0XHF4.pdb_B',
       'A0A0P0XHI5.pdb_B', 'A0A0P0XNQ3.pdb_B', 'A0A0P0XSL8.pdb_B',
       'A0A0P0Y2U6.pdb_B', 'A0A0P0Y5S9.pdb_B', 'A0A0P0YA59.pdb_B',
       'A0A0P0YAA2.pdb_B', 'A0A0P0YC15.pdb_B', 'A2ZPC4.pdb_B',
       'B7ESQ3.pdb_B', 'B9G6M6.pdb_B', 'Q0DQG8.pdb_B', 'Q0JB10.pdb_B',
       'Q0JK63.pdb_B', 'Q0JPJ6.pdb_B', 'Q109D8.pdb_B', 'Q2QQH8.pdb_B',
       'Q6ZDH1.pdb_B', 'A0A0N7KD81.pdb_A', 'A0A0N7KK85.pdb_B',
       'A0A0N7KN01.pdb_B', 'A0A0N7KT42.pdb_B', 'A0A0P0V091.pdb_B',
       'A0A0P0V3R7.pdb_B', 'A0A0P0WDY8.pdb_B', 'A0A0P0WQD9.pdb_B',
       'A0A0P0X3V6.pdb_B', 'A0A0P0X458.pdb_B', 'Q5N762.pdb_B',
       'Q5Z7U2.pdb_B', 'Q69J07.pdb_B', 'Q69X07.pdb_B', 'Q75IQ5.pdb_B',
       'Q75J50.pdb_B', 'Q7EY32.pdb_B', 'Q7FAH1.pdb_B', 'Q7XAK4.pdb_B',
       'Q8LJA9.pdb_B'], dtype=object)
```

Print the PATHs of the subcluster representatives to open them in PyMOL:

In [ ]:
```
' '.join([path_cluster + str(results_dir) + f'/merged_clusters/cluster_{clus
```

Write down the subclusters in different categories:

Binding motif --- A0A0P0VGE5 A0A0P0VHD9 A0A0P0VHP5 A0A0P0VNF2 A0A0P0VNI4 A0A0P0X4D4 A0A0P0XCY3 A0A0P0XE00 A0A0P0XE77 A0A0P0XEW7 A0A0P0XHF4 A0A0P0XHI5 A0A0P0XNQ3 A0A0P0XSL8 A0A0P0YA59 A0A0P0YAA2 B7ESQ3 B9G6M6 Q109D8 Q2QQH8 A0A0N7KK85 A0A0N7KT42 A0A0P0X458 Q5N762 Q69J07 Q69X07 Q75IQ5 Q7EY32 Q7XAK4 Horseshoe --- A0A0P0VRN2 A0A0P0XFT5 A0A0P0Y2U6 A0A0P0Y5S9 A0A0P0YC15 A0A0N7KD81 A0A0P0V091 A0A0P0WQD9 A0A0P0X3V6 Q5Z7U2 Q75J50 Q7FAH1 Beta propeller --- A0A0P0W419 Q0DQG8 Q0JK63 Q6ZDH1 A0A0P0V3R7 Beta other --- A0A0P0VZH9 A2ZPC4 Q0JB10 Q0JPJ6 A0A0N7KN01 A0A0P0WDY8 Q8LJA9

Subclusters for only the binding motif

In [ ]:
```
subcluster_names = [
'A0A0P0VGE5.pdb_B',
'A0A0P0VHD9.pdb_B',
```

```
'A0A0P0VHP5.pdb_B',
'A0A0P0VNF2.pdb_B',
'A0A0P0VNI4.pdb_B',
'A0A0P0X4D4.pdb_B',
'A0A0P0XCY3.pdb_B',
'A0A0P0XE00.pdb_B',
'A0A0P0XE77.pdb_B',
'A0A0P0XEW7.pdb_B',
'A0A0P0XHF4.pdb_B',
'A0A0P0XHI5.pdb_B',
'A0A0P0XNQ3.pdb_B',
'A0A0P0XSL8.pdb_B',
'A0A0P0YA59.pdb_B',
'A0A0P0YAA2.pdb_B',
'B7ESQ3.pdb_B',
'B9G6M6.pdb_B',
'Q109D8.pdb_B',
'Q2QQH8.pdb_B',
'A0A0N7KK85.pdb_B',
'A0A0N7KT42.pdb_B',
'A0A0P0X458.pdb_B',
'Q5N762.pdb_B',
'Q69J07.pdb_B',
'Q69X07.pdb_B',
'Q75IQ5.pdb_B',
'Q7EY32.pdb_B',
'Q7XAK4.pdb_B'
]
```

See how many proteins are in these subclusters:

In [ ]:
```
(clustered_clusters[clustered_clusters.subcluster_rep.isin(subcluster_names)
 .subcluster_rep.value_counts().sum())
```

Out[ ]: 113

See the alignment scores of the top members in these subclusters:

In [ ]:
```
# Get list of members for subcluster
members_sc = list(clustered_clusters[clustered_clusters.subcluster_rep.isin(
                  .member)

median_scores[median_scores.member.isin(members_sc)].sort_values(by='rmsd').
```

Out[ ]:

	cluster	member	tmscore	rmsd	aligned_length	cluster_size
**19**	A0A0P0XHF4.pdb_B	A0A0N7KK85	0.941210	1.530	210.0	695.0
**520**	A0A0P0XHF4.pdb_B	Q6ASY4	0.938210	1.560	213.0	695.0
**401**	A0A0P0XHF4.pdb_B	Q2QQH8	0.938700	1.600	215.0	695.0
**37**	A0A0P0XHF4.pdb_B	A0A0N7KT42	0.935435	1.630	208.0	695.0
**256**	A0A0P0XHF4.pdb_B	A0A0P0YAQ4	0.935185	1.645	217.0	695.0
**700**	A0A0P0XHF4.pdb_B	Q9FWD4	0.923980	1.740	216.0	695.0
**418**	A0A0P0XHF4.pdb_B	Q2R0S7	0.934600	1.760	217.0	695.0
**145**	A0A0P0XHF4.pdb_B	A0A0P0X0M4	0.921310	1.780	215.0	695.0
**290**	A0A0P0XHF4.pdb_B	B9FCI9	0.919485	1.830	219.0	695.0
**394**	A0A0P0XHF4.pdb_B	Q2QMT0	0.918475	1.830	216.0	695.0
**88**	A0A0P0XHF4.pdb_B	A0A0P0W0W5	0.933120	1.840	218.0	695.0
**14**	A0A0P0XHF4.pdb_B	A0A0N7KHR2	0.932530	1.850	218.0	695.0
**480**	A0A0P0XHF4.pdb_B	Q5VNR1	0.922670	1.850	216.0	695.0
**333**	A0A0P0XHF4.pdb_B	Q0ITY1	0.917245	1.855	206.0	695.0
**693**	A0A0P0XHF4.pdb_B	Q943F5	0.924290	1.860	213.0	695.0
**361**	A0A0P0XHF4.pdb_B	Q0JI48	0.924270	1.870	218.0	695.0
**71**	A0A0P0XHF4.pdb_B	A0A0P0VLY9	0.923905	1.880	217.0	695.0
**232**	A0A0P0XHF4.pdb_B	A0A0P0XZS4	0.921440	1.910	218.0	695.0
**157**	A0A0P0XHF4.pdb_B	A0A0P0X4D4	0.915655	1.915	212.0	695.0
**254**	A0A0P0XHF4.pdb_B	A0A0P0YAA2	0.914570	1.940	211.0	695.0

See if any of the homologous proteins are in here:

In [ ]:
```
[h in members_sc for h in homologues]
```

Out[ ]: [False, False, False, True, False]

In [ ]:
```
homologues
```

Out[ ]: ['Q5VMP0', 'A0A0N7KEW0', 'Q8RZQ3', 'Q69X07', 'A0A0P0Y6A8']

`Q69X07` is in this subcluster.

Print the paths of the top 20 PDB files to open them in PyMOL:

In [ ]:
```
# Print paths to open models in pymol
membs = median_scores[median_scores.member.isin(members_sc)].sort_values(by=
' '.join([(path_cluster + str(results_dir) + f'/merged_clusters/cluster_{clu
```

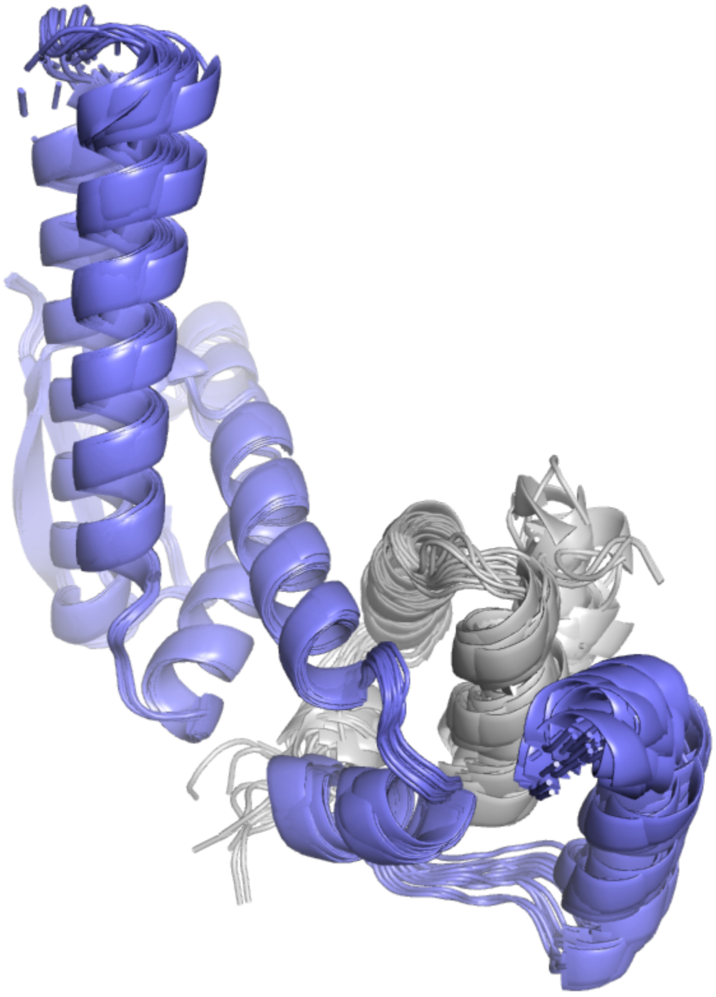

This is very similar to the previous figure with all the subclusters together. The models with the lowest RMSD overall are those who only contain the minimal bindign motif of the binder protein, since they have fewer residues to align.

Subclusters for the binding motif + Leucine-rich region (horseshoe)

In [ ]:
```
subcluster_names = [
'A0A0P0VRN2.pdb_B',
'A0A0P0XFT5.pdb_B',
'A0A0P0Y2U6.pdb_B',
'A0A0P0Y5S9.pdb_B',
'A0A0P0YC15.pdb_B',
'A0A0N7KD81.pdb_B',
```

```
'A0A0P0V091.pdb_B',
'A0A0P0WQD9.pdb_B',
'A0A0P0X3V6.pdb_B',
'Q5Z7U2.pdb_B',
'Q75J50.pdb_B',
'Q7FAH1.pdb_B'
]
```

See how many proteins are in these subclusters:

In [ ]:
```
(clustered_clusters[clustered_clusters.subcluster_rep.isin(subcluster_names)
 .subcluster_rep.value_counts().sum())
```

Out[ ]: 262

See the alignment scores of the top members in these subclusters:

In [ ]:
```
# Get list of members for subcluster
members_sc = list(clustered_clusters[clustered_clusters.subcluster_rep.isin(
                  .member)

median_scores[median_scores.member.isin(members_sc)].sort_values(by='rmsd').
```

Out[ ]:

	cluster	member	tmscore	rmsd	aligned_length	cluster_size
**171**	A0A0P0XHF4.pdb_B	A0A0P0XBZ2	0.687465	7.325	230.5	695.0
**149**	A0A0P0XHF4.pdb_B	A0A0P0X2K4	0.415300	7.340	216.0	695.0
**82**	A0A0P0XHF4.pdb_B	A0A0P0VUW0	0.711865	7.670	270.0	695.0
**413**	A0A0P0XHF4.pdb_B	Q2QY97	0.432800	7.685	221.0	695.0
**81**	A0A0P0XHF4.pdb_B	A0A0P0VRP6	0.596455	7.750	221.5	695.0
**310**	A0A0P0XHF4.pdb_B	C7J8M0	0.600700	7.785	219.0	695.0
**118**	A0A0P0XHF4.pdb_B	A0A0P0WF72	0.641515	7.825	238.0	695.0
**282**	A0A0P0XHF4.pdb_B	B7EXZ6	0.514025	8.010	214.0	695.0
**249**	A0A0P0XHF4.pdb_B	A0A0P0Y5S9	0.551080	8.015	209.0	695.0
**244**	A0A0P0XHF4.pdb_B	A0A0P0Y2Y9	0.417340	8.035	218.5	695.0
**367**	A0A0P0XHF4.pdb_B	Q0JLI9	0.285820	8.055	223.0	695.0
**359**	A0A0P0XHF4.pdb_B	Q0JES5	0.269000	8.165	217.5	695.0
**366**	A0A0P0XHF4.pdb_B	Q0JLI0	0.276000	8.175	220.0	695.0
**69**	A0A0P0XHF4.pdb_B	A0A0P0VJA5	0.375275	8.200	230.0	695.0
**248**	A0A0P0XHF4.pdb_B	A0A0P0Y4Y4	0.704520	8.275	242.5	695.0
**647**	A0A0P0XHF4.pdb_B	Q7XSL8	0.264890	8.320	222.0	695.0
**34**	A0A0P0XHF4.pdb_B	A0A0N7KR34	0.508930	8.380	215.0	695.0
**358**	A0A0P0XHF4.pdb_B	Q0JES4	0.275240	8.555	218.5	695.0
**533**	A0A0P0XHF4.pdb_B	Q6ETW1	0.453605	8.555	222.5	695.0
**667**	A0A0P0XHF4.pdb_B	Q8GVM0	0.290510	8.570	220.5	695.0

See if any of the homologous proteins are in here:

In [ ]:
```
[h in members_sc for h in homologues]
```

Out[ ]:
```
[True, True, True, False, True]
```

In [ ]:
```
homologues
```

Out[ ]:
```
['Q5VMP0', 'A0A0N7KEW0', 'Q8RZQ3', 'Q69X07', 'A0A0P0Y6A8']
```

All the rest of homologous proteins are in here!

Print the paths of the top 20 PDB files to open them in PyMOL:

In [ ]:
```
# Print paths to open models in pymol
membs = median_scores[median_scores.member.isin(members_sc)].sort_values(by=
' '.join([(path_cluster + str(results_dir) + f'/merged_clusters/cluster_{clu
```

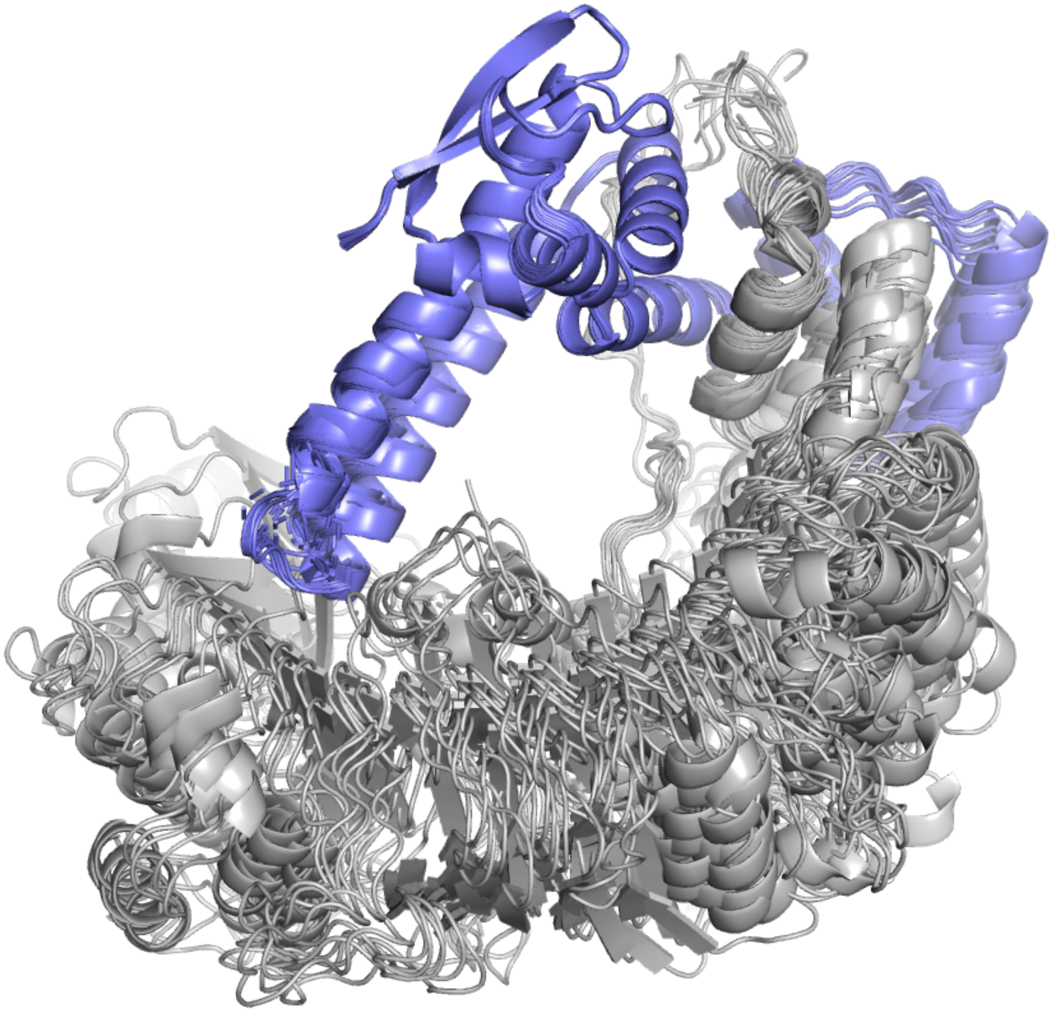

These subclusters contain binders that, in addition to the binding motif, have LLR domains that also interact with SKP1 in some way. The true binder of SKP1 and its homologues have a similar architecture.

Subclusters for the binding motif + beta propeller

In [ ]:
```
subcluster_names = [
'A0A0P0W419.pdb_B',
'Q0DQG8.pdb_B',
'Q0JK63.pdb_B',
'Q6ZDH1.pdb_B',
'A0A0P0V3R7.pdb_B'
]
```

See how many proteins are in these subclusters:

In [ ]:
```
(clustered_clusters[clustered_clusters.subcluster_rep.isin(subcluster_names)
 .subcluster_rep.value_counts().sum())
```

Out[ ]: 277

See the alignment scores of the top members in these subclusters:

In [ ]:
```
# Get list of members for subcluster
members_sc = list(clustered_clusters[clustered_clusters.subcluster_rep.isin(
                  .member)

median_scores[median_scores.member.isin(members_sc)].sort_values(by='rmsd').
```

Out[ ]:

	cluster	member	tmscore	rmsd	aligned_length	cluster_size
**213**	A0A0P0XHF4.pdb_B	A0A0P0XQK8	0.302510	7.940	201.5	695.0
**133**	A0A0P0XHF4.pdb_B	A0A0P0WR08	0.309095	8.335	188.0	695.0
**422**	A0A0P0XHF4.pdb_B	Q2R1S8	0.293405	8.740	194.0	695.0
**528**	A0A0P0XHF4.pdb_B	Q6EQC5	0.351750	8.760	197.5	695.0
**575**	A0A0P0XHF4.pdb_B	Q6Z4S1	0.318150	8.760	206.5	695.0
**691**	A0A0P0XHF4.pdb_B	Q8VWI8	0.295920	8.865	194.0	695.0
**360**	A0A0P0XHF4.pdb_B	Q0JFD5	0.274020	8.890	191.0	695.0
**692**	A0A0P0XHF4.pdb_B	Q8W0I3	0.308530	8.955	195.0	695.0
**471**	A0A0P0XHF4.pdb_B	Q53WL8	0.333390	9.060	197.0	695.0
**577**	A0A0P0XHF4.pdb_B	Q6Z6Y9	0.307905	9.070	197.0	695.0
**66**	A0A0P0XHF4.pdb_B	A0A0P0VI66	0.302140	9.100	194.0	695.0
**129**	A0A0P0XHF4.pdb_B	A0A0P0WQ78	0.293750	9.125	191.0	695.0
**599**	A0A0P0XHF4.pdb_B	Q6ZK55	0.313985	9.145	198.0	695.0
**578**	A0A0P0XHF4.pdb_B	Q6Z7A7	0.333375	9.185	197.0	695.0
**513**	A0A0P0XHF4.pdb_B	Q69RN5	0.279535	9.300	192.0	695.0
**474**	A0A0P0XHF4.pdb_B	Q5QM20	0.326920	9.310	213.0	695.0
**128**	A0A0P0XHF4.pdb_B	A0A0P0WPS2	0.290210	9.330	195.0	695.0
**267**	A0A0P0XHF4.pdb_B	A0A5S6R9A0	0.329460	9.330	209.0	695.0
**677**	A0A0P0XHF4.pdb_B	Q8LHX9	0.326415	9.380	202.5	695.0
**363**	A0A0P0XHF4.pdb_B	Q0JKP0	0.297210	9.410	195.0	695.0

Print the paths of the top 20 PDB files to open them in PyMOL:

In [ ]:
```
# Print paths to open models in pymol
membs = median_scores[median_scores.member.isin(members_sc)].sort_values(by=
' '.join([(path_cluster + str(results_dir) + f'/merged_clusters/cluster_{clu
```

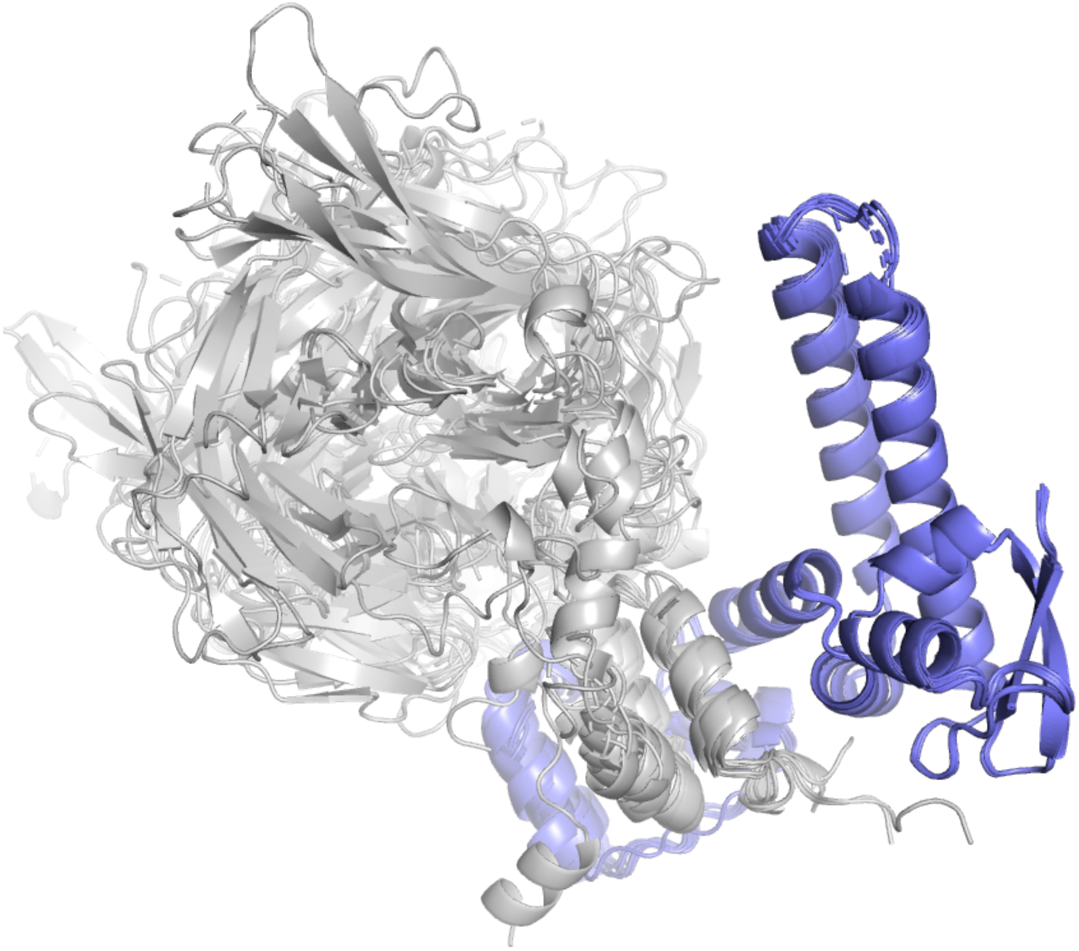

The alignment of the beta propeller domains is much messier, as they are modeled in many different orientations. The screenshot above only contains a few of the complexes.

In [ ]:

Clustering AVR-Pia

This notebook will guide you through the process of clustering 99 complexes of the AVR-Pia protein against a sample of rice proteins. All of the models are dimeric. The 99 models originate from a proteome-wide screen of AVR-Pia against the rice proteome (O. sativa subsp. japonica, 43,000+ initial models). To select this sample we first ran AlphaCRV on all the models, and the 99 structures are part of some of the best clusters that were identified. Running the pipeline on these models should be enough to reproduce the results and give you an idea of the workflow.

Prerequisites

- Install AlphaCRV on a conda environment and activate it
- Download the sequences, models and results for this example at Zenodo

1. Cluster the models with the `clustering` command

Run the following command to cluster the models. Make sure to change the paths according to your system:

```
alphacrv-cluster \
  --bait ./examples/AVRPia/AVRPia.fasta \
  --binders ./examples/AVRPia/AVRPia_binders.fasta \
  --models_dir ./examples/AVRPia/AVRPia_vs_rice_models \
  --destination ./examples/AVRPia/AVRPia_vs_rice_clusters \
  --cpus 8
```

After collecting the quality scores from the models in `--models_dir`, it will count how many models are there with an ipTM scores higher than the threshold (0.75 by default). It will prompt you to confirm or to modify the threshold. After that, it will proceed with the clustering.

The full output is as follows:

```
(env) AlphaCRV$ alphacrv-cluster \
--bait ./examples/AVRPia/AVRPia.fasta \
--binders ./examples/AVRPia/AVRPia_binders.fasta \
--models_dir ./examples/AVRPia/AVRPia_vs_rice_models \
--destination ./examples/AVRPia/AVRPia_vs_rice_clusters \
--cpus 8
INFO:root:Getting quality scores for models in
examples/AVRPia/AVRPia_vs_rice_models...
INFO:root:Found 99 model directories with quality scores.
Will select 99 models with iptm >= 0.75. Press enter to
continue, or enter a new threshold:
INFO:root:Trimming binder molecules to keep only regions with
an average PAE against the bait of up to 10.0...
INFO:root:Processed 99 complexes.
INFO:root:Writing trimmed sequences to fasta file.
INFO:root:Running sequence clustering...
INFO:root:Processing output...
INFO:root:Running structural clustering...
INFO:root:Processing output...
INFO:root:Aligning all vs all members of each cluster...
INFO:root:Aligning cluster 1 of 3...
INFO:root:49 members.
INFO:root:Aligning cluster 2 of 3...
INFO:root:40 members.
INFO:root:Aligning cluster 3 of 3...
INFO:root:10 members.
INFO:root:Calculating median alignment scores...
INFO:root:Done!!
```

After this step you will have a directory with the following structure:

```
(env) AlphaCRV$ ll examples/AVRPia/AVRPia_vs_rice_clusters/
total 36K
-rw-r--r-- 1 example g-example 2.0K Jan  3 19:39
binders_regions.csv
drwxr-xr-x 1 example g-example    0 Jan  3 19:40
merged_clusters/
drwxr-xr-x 1 example g-example    0 Jan  3 19:39
pdbs_trimmed/
drwxr-xr-x 1 example g-example    0 Jan  3 19:39 seqclusters/
drwxr-xr-x 1 example g-example    0 Jan  3 19:39 strclusters/
-rw-r--r-- 1 example g-example  29K Jan  3 19:39
trimmed_binders.fasta
```

Now let's look at some of the important files:

In [ ]:
```
from pathlib import Path
import pandas as pd
```

In [ ]:
```
results_dir = Path('./AVRPia/AVRPia_vs_rice_clusters/')
```

See clusters

The `merged_clusters.csv` file contains the list of models with their corresponding sequence, structure and merged clusters. It also has the quality scores provided by AlphaFold.

In [ ]:
```
clusters = pd.read_csv(results_dir / 'merged_clusters/merged_clusters.csv')
```

In [ ]:
```
clusters
```

Out[ ]:

	complex	str_rep	seq_rep	merged_rep	member
**0**	6Q76-1_Q5JN27-1	Q0IYV8.pdb_B	C7IWS6	Q6Z1A9.pdb_B	Q5JN27
**1**	6Q76-1_Q6K9S6-1	A0A0P0WCR3.pdb_B	Q6K9S6	Q6Z1A9.pdb_B	Q6K9S6
**2**	6Q76-1_A0A0P0VEX0-1	Q0IYV8.pdb_B	A0A0P0XZP1	Q6Z1A9.pdb_B	A0A0P0VEX0
**3**	6Q76-1_Q2R3L3-1	A0A0P0VF33.pdb_B	A0A0P0Y2U8	Q6Z1A9.pdb_B	Q2R3L3
**4**	6Q76-1_A0A0P0UZA6-1	A0A0P0UZA6.pdb_B	A0A0P0UZA6	Q7XL74.pdb_B	A0A0P0UZA6
**...**	...	...	...	...	...
**94**	6Q76-1_A0A0P0XDF8-1	Q0IYV8.pdb_B	A0A0N7KMC1	Q6Z1A9.pdb_B	A0A0P0XDF8
**95**	6Q76-1_A0A0P0Y3K7-1	Q0IYV8.pdb_B	A0A0P0Y3K7	Q6Z1A9.pdb_B	A0A0P0Y3K7
**96**	6Q76-1_Q0IYI4-1	Q7XL74.pdb_B	Q7XL74	Q7XL74.pdb_B	Q0IYI4
**97**	6Q76-1_Q6Z0A9-1	Q0IYV8.pdb_B	A0A0N7KMC1	Q6Z1A9.pdb_B	Q6Z0A9
**98**	6Q76-1_Q84TS2-1	Q7XL74.pdb_B	Q8S181	Q7XL74.pdb_B	Q84TS2

99 rows × 7 columns

The columns of the `clusters` DataFrame are:

- `complex` : The name of the complex. This is the same name as the directory where the model is stored.
- `str_rep` : Name of the structure cluster representative
- `seq_rep` : Name of the sequence cluster representative
- `merged_rep` : Name of the merged cluster representative (sequence + structure)
- `member` : The ID of the binder protein
- `iptm` : The ipTM score of the model
- `iptm+ptm` : The ipTM+PTM score of the model (it is calculated by AlphaFold as $0.8 ipTM + 0.2$pTM)

See the number of different merged clusters:

In [ ]:
```
clusters.merged_rep.unique().shape
```

Out[ ]: (3,)

For this example, the models and sequences from the 99 binder proteins were summarized in 3 clusters. Much fewer structures to sort through!

See alignment scores

Alignment scores are calcualted for each cluster by aligning all vs all members of the cluster.

In [ ]:
```
alignment_scores = pd.read_csv(results_dir / 'merged_clusters/alignment_scor
```

In [ ]:
```
alignment_scores.head()
```

Out[ ]:

	cluster	ref	member	tmscore_ref	tmscore_m	aligned_length	rmsd
**0**	Q6Z1A9.pdb_B	Q5JN27	Q6K9S6	0.22351	0.56157	206	12.72
**1**	Q6Z1A9.pdb_B	Q5JN27	A0A0P0VEX0	0.53580	0.44881	564	20.63
**2**	Q6Z1A9.pdb_B	Q5JN27	Q2R3L3	0.32890	0.66186	274	10.16
**3**	Q6Z1A9.pdb_B	Q5JN27	A0A0P0Y2U8	0.23944	0.51955	225	11.05
**4**	Q6Z1A9.pdb_B	Q5JN27	C7IWS6	0.55887	0.81560	393	9.61

The columns of the `alignment_scores` DataFrame are:

- `cluster` : The name of the cluster
- `ref` : Binder ID of the reference structure in the alignment
- `member` : Binder ID of the second structure in the alignment
- `tmscore_ref` : TM-score based on the reference structure
- `tmscore_m` : TM-score based on the second structure
- `aligned_length` : Length of the alignment
- `rmsd` : RMSD of the alignment

Based on these scores, the median scores are calculated for each cluster member to find the best representative of the cluster (the one with lowest RMSD score to the other members).

Read median scores and find top clusters

Now we can rank the clusters based on the median alignment scores of the cluster representatives:

In [ ]:
```
median_scores = pd.read_csv(results_dir / 'merged_clusters/median_scores.csv
```

In [ ]:
```
median_scores.shape
```

Out[ ]: (99, 7)

The `median_scores` DataFrame contains the median alignment scores of each cluster member when aligned to all other members of the same cluster.

In [ ]:
```
median_scores.head()
```

Out[ ]:

	cluster	member	tmscore	rmsd	aligned_length	cluster_size	fraction_binder
**0**	P42211.pdb_B	A0A0P0VAB8	0.56928	21.55	419.0	10.0	0.862408
**1**	P42211.pdb_B	A0A0P0VAW6	0.59934	12.81	407.0	10.0	0.867008
**2**	P42211.pdb_B	A0A0P0WVN9	0.39349	24.73	412.0	10.0	0.577181
**3**	P42211.pdb_B	P42211	0.26818	20.91	363.0	10.0	0.707434
**4**	P42211.pdb_B	Q0D5V1	0.66389	10.75	402.0	10.0	0.902703

The columns of the `median_scores` DataFrame are:

- `cluster` : The name of the cluster
- `member` : ID of the cluster member (binder molecule)
- `tmscore` : Median TM-score of the complex against all other complexes in this cluster
- `rmsd` : Median RMSD of the complex against all other complexes in this cluster
- `aligned_length` : Median length of the alignment
- `cluster_size`
- `fraction_binder` : In average, how much of the binder molecule is included in the alignments of this complex against all other complexes (calculated as `(aligned_length - bait_length)/binder_length`). This is just meant to be an approximation of how complete the aligmnents are for this cluster member.

The next step is to select the cluster representatives. For this, we first need to filter out the cluster members with poor quality alignments, according to the following criteria:

- Small size
- Low median TM-score
- High median RMSD
- Low fraction of the binder aligned in the cluster representative

In [ ]:
```
# Select the clusters with the following criteria:
select = ((median_scores.cluster_size >= 5) & \
          (median_scores.tmscore >= 0.2) & \
          (median_scores.fraction_binder >= 0.2) & \
          (median_scores.rmsd <= 15))
median_scores_filtered = median_scores[select]
```

In [ ]:
```
median_scores_filtered.shape
```

Out[ ]: (83, 7)

See how many clusters are left after filtering:

In [ ]:
```
median_scores_filtered.cluster.unique().shape
```

Out[ ]: (3,)

Function to format tables:

In [ ]:
```
import seaborn as sns
cm_r = sns.color_palette("mako_r", as_cmap=True)
cm = sns.color_palette("mako", as_cmap=True)
```

In [ ]:
```
def make_pretty(styler):
    styler.format(precision=2)
    styler.background_gradient(axis=0, cmap=cm_r, subset=pd.IndexSlice[:,"cl
    styler.background_gradient(axis=0, cmap=cm_r, subset=pd.IndexSlice[:,"tm
    styler.background_gradient(axis=0, cmap=cm, subset=pd.IndexSlice[:,"rmsd
    styler.background_gradient(axis=0, cmap=cm_r, subset=pd.IndexSlice[:,"fr
    return styler
```

RESULT 1: See clusters ranked by RMSD

Finally, we can rank the clusters and see which ones have a good combination of low RMSD and large cluster size. These ones are the most likely to contain the true binder.

In [ ]:
```
# Select the rows with the minimum RMSD for each cluster
select = median_scores_filtered.groupby('cluster').rmsd.idxmin()
columns = ['cluster', 'tmscore', 'rmsd', 'cluster_size', 'fraction_binder']
median_scores_filtered.loc[select,columns].sort_values(by='rmsd').style.pipe
```

Out[ ]:

	cluster	tmscore	rmsd	cluster_size	fraction_binder
**59**	Q7XL74.pdb_B	0.90	1.49	40.00	0.94
**22**	Q6Z1A9.pdb_B	0.55	10.65	49.00	0.84
**4**	P42211.pdb_B	0.66	10.75	10.00	0.90

Here we can see that the cluster `Q7XL74.pdb_B` has the lowest median RMSD and the highest median TM-score. It also has a very large size with 40 members.

RESULT 2: See clusters ranked by size

In [ ]:
```
# Select the rows with the minimum RMSD for each cluster
select = median_scores_filtered.groupby('cluster').rmsd.idxmin()
columns = ['cluster', 'tmscore', 'rmsd', 'cluster_size', 'fraction_binder']
median_scores_filtered.loc[select, columns].sort_values(by='cluster_size', a
```

Out[ ]:

	cluster	tmscore	rmsd	cluster_size	fraction_binder
22	Q6Z1A9.pdb_B	0.55	10.65	49.00	0.84
59	Q7XL74.pdb_B	0.90	1.49	40.00	0.94
4	P42211.pdb_B	0.66	10.75	10.00	0.90

Here we can see that the cluster `Q6Z1A9.pdb_B` is the largest cluster with 49 members, but it has a much worse RMSD than `Q7XL74.pdb_B`. It would be interesting to look at both and see how they compare. So we managed to reduce more than 43,000 starting models to only two promising clusters!

For this example we know that in our list of candidate binders there are 14 homologues of the true binder protein. We can find out which clusters contain these homologues:

In [ ]:

```
homologues = ['Q6YY33', 'Q6YY34', 'A0A0P0VKX7', 'Q0JCK8', 'Q6YY31', 'Q7XJV3'
        'A0A0N7KFK3', 'Q7XJV0', 'A0A0P0WB87', 'Q6EPT4', 'Q0J314', 'Q6EPT2',
        'Q8S5W0', 'Q2QSQ7']
```

In [ ]:

```
clusters[clusters.member.isin(homologues)][['complex','merged_rep','iptm','i
```

Out[ ]:

	complex	merged_rep	iptm	iptm+ptm
6	6Q76-1_Q6EPT2-1	Q7XL74.pdb_B	0.913843	0.847186
14	6Q76-1_Q6EPT4-1	Q7XL74.pdb_B	0.903790	0.819935
26	6Q76-1_Q2QSQ7-1	Q7XL74.pdb_B	0.892084	0.806683
30	6Q76-1_Q0J314-1	Q7XL74.pdb_B	0.886320	0.820720
38	6Q76-1_Q8S5W0-1	Q7XL74.pdb_B	0.875703	0.805274
43	6Q76-1_Q6YY34-1	Q7XL74.pdb_B	0.867315	0.839418
47	6Q76-1_Q7XJV3-1	Q7XL74.pdb_B	0.862152	0.831752
48	6Q76-1_Q6YY33-1	Q7XL74.pdb_B	0.860912	0.830552
55	6Q76-1_A0A0N7KFK3-1	Q7XL74.pdb_B	0.857097	0.819887
57	6Q76-1_A0A0P0VKX7-1	Q7XL74.pdb_B	0.854087	0.827703
63	6Q76-1_A0A0P0WB87-1	Q7XL74.pdb_B	0.843057	0.768634
71	6Q76-1_Q6YY31-1	Q7XL74.pdb_B	0.825141	0.805893
76	6Q76-1_Q0JCK8-1	Q7XL74.pdb_B	0.818324	0.793344
84	6Q76-1_Q7XJV0-1	Q7XL74.pdb_B	0.806434	0.778532

They are all in the top cluster by RMSD!

2. Make pymol sessions for the top clusters with `make_pymol_sessions`

Run the following command to select the top clusters that we saw above, make pymol sessions of the top clusters, and optionally do structural clustering on each cluster to find subclusters:

```
alphacrv-rank \
  --clusters_dir ./examples/AVRPia/AVRPia_vs_rice_clusters \
  --min_members 5 \
  --min_tmscore 0.2 \
  --max_rmsd 15 \
  --cluster_clusters
```

The program will show you the top clusters that will be used to make the pymol sessions. You can press `Enter` to continue, or exit the program with `Ctrl+C` to change the filtering parameters.

```
(env) AlphaCRV$ alphacrv-rank \
--clusters_dir ./examples/AVRPia/AVRPia_vs_rice_clusters \
--min_members 5 \
--min_tmscore 0.2 \
--max_rmsd 15 \
--cluster_clusters
INFO:root:Identified 3 top clusters.
INFO:root:Top clusters:

        cluster  tmscore    rmsd  cluster_size
fraction_binder
0  Q6Z1A9.pdb_B  0.54957  10.655          49.0
0.839820
1  Q7XL74.pdb_B  0.89660   1.490          40.0
0.935897
2  P42211.pdb_B  0.66389  10.750          10.0
0.902703

Press Enter to continue, or Ctrl+C to exit and select
different filtering parameters:
INFO:root:Copying pdbs from the top clusters...
INFO:root:Making Pymol sessions...
PyMOL>select chain B AND model P42211_repB
 Selector: selection "sele" defined with 6294 atoms.
PyMOL>bg white
PyMOL>set ray_shadow, 0
 Setting: ray_shadow set to off.
PyMOL>color grey80
 Executive: Colored 72547 atoms.
PyMOL>select chain A
 Selector: selection "sele" defined with 10530 atoms.
PyMOL>color slate, sele
 Executive: Colored 10530 atoms.
PyMOL>delete all
PyMOL>select chain B AND model Q6Z1A9_repB
 Selector: selection "sele" defined with 3855 atoms.
PyMOL>bg white
PyMOL>set ray_shadow, 0
 Setting: ray_shadow set to off.
PyMOL>color grey80
 Executive: Colored 368033 atoms.
PyMOL>select chain A
 Selector: selection "sele" defined with 51597 atoms.
PyMOL>color slate, sele
 Executive: Colored 51597 atoms.
PyMOL>delete all
PyMOL>select chain B AND model Q7XL74_repB
 Selector: selection "sele" defined with 1365 atoms.
PyMOL>bg white
PyMOL>set ray_shadow, 0
 Setting: ray_shadow set to off.
```

```
PyMOL>color grey80
 Executive: Colored 92930 atoms.
PyMOL>select chain A
 Selector: selection "sele" defined with 42120 atoms.
PyMOL>color slate, sele
 Executive: Colored 42120 atoms.
PyMOL>delete all
INFO:root:Clustering clusters...
INFO:root:Clustering P42211.pdb_B
INFO:root:Running structural clustering...
INFO:root:Processing output...
INFO:root:Clustering Q6Z1A9.pdb_B
INFO:root:Running structural clustering...
INFO:root:Processing output...
INFO:root:Clustering Q7XL74.pdb_B
INFO:root:Running structural clustering...
INFO:root:Processing output...
INFO:root:Done!!
```

This command should create the following files in the `./examples/AVRPia/AVRPia_vs_rice_clusters/merged_clusters/` / `merged_clusters` directory:

- `clustered_clusters.csv` : Contains the subclusters for each of the top clusters.
- `cluster_<cluster_ID>/` : Contains the PDBs of each cluster, and a PyMol session with the cluster members.
- `cluster_<cluster_ID>_clusters/` : Contains the results of the `foldseek easy-cluster` run on the cluster members.

Read clustered clusters

The following DataFrame contains the subclusters for each of the top clusters:

In [ ]:
```
clustered_clusters = pd.read_csv(results_dir / 'merged_clusters/clustered_cl
```

In [ ]:
```
clustered_clusters.head()
```

Out[ ]:

	subcluster_rep	member	cluster
**0**	P42211.pdb_B	P42211	P42211.pdb_B
**1**	P42211.pdb_B	Q6F4N5	P42211.pdb_B
**2**	P42211.pdb_B	A0A0P0VAB8	P42211.pdb_B
**3**	P42211.pdb_B	Q0D5V1	P42211.pdb_B
**4**	P42211.pdb_B	Q8LNM1	P42211.pdb_B

Now we can look at the most interesting clusters and their subclusters in more detail:

RESULT 1: Cluster Q7XL74.pdb_B (contains true binder homologs, top cluster by size)

In [ ]:
```
cluster = 'Q7XL74.pdb_B'
```

See subclusters:

In [ ]:
```
clustered_clusters[clustered_clusters.cluster==cluster]
```

Out[ ]:

	subcluster_rep	member	cluster
**59**	A0A0P0UZA6.pdb_B	A0A0P0UZA6	Q7XL74.pdb_B
**60**	Q7XL74.pdb_B	Q7XL74	Q7XL74.pdb_B
**61**	Q7XL74.pdb_B	Q0IYI4	Q7XL74.pdb_B
**62**	Q7XL74.pdb_B	Q75J02	Q7XL74.pdb_B
**63**	Q7XL74.pdb_B	A0A0P0WEB4	Q7XL74.pdb_B
**64**	Q7XL74.pdb_B	A0A0P0VRA1	Q7XL74.pdb_B
**65**	Q7XL74.pdb_B	Q0JBG2	Q7XL74.pdb_B
**66**	Q7XL74.pdb_B	A0A0P0XD00	Q7XL74.pdb_B
**67**	Q7XL74.pdb_B	A0A0P0V126	Q7XL74.pdb_B
**68**	Q7XL74.pdb_B	A3AYA2	Q7XL74.pdb_B
**69**	Q7XL74.pdb_B	Q10KH9	Q7XL74.pdb_B
**70**	Q7XL74.pdb_B	Q84TS2	Q7XL74.pdb_B
**71**	Q7XL74.pdb_B	Q8LNN6	Q7XL74.pdb_B
**72**	Q7XL74.pdb_B	Q8S181	Q7XL74.pdb_B
**73**	Q7XL74.pdb_B	Q7G2B2	Q7XL74.pdb_B
**74**	Q7XL74.pdb_B	A0A0P0XFR7	Q7XL74.pdb_B
**75**	Q7XL74.pdb_B	A3ADD6	Q7XL74.pdb_B
**76**	Q7XL74.pdb_B	Q6I5G4	Q7XL74.pdb_B
**77**	Q7XL74.pdb_B	Q6YY31	Q7XL74.pdb_B
**78**	Q7XL74.pdb_B	Q84TB9	Q7XL74.pdb_B
**79**	Q7XL74.pdb_B	Q8LN41	Q7XL74.pdb_B
**80**	Q7XL74.pdb_B	B7E663	Q7XL74.pdb_B
**81**	Q7XL74.pdb_B	A0A0N7KFK3	Q7XL74.pdb_B
**82**	Q7XL74.pdb_B	A0A0P0VKX7	Q7XL74.pdb_B
**83**	Q7XL74.pdb_B	A0A0P0WB87	Q7XL74.pdb_B
**84**	Q7XL74.pdb_B	Q0JCK8	Q7XL74.pdb_B
**85**	Q7XL74.pdb_B	Q6EPT2	Q7XL74.pdb_B
**86**	Q7XL74.pdb_B	Q6YY33	Q7XL74.pdb_B
**87**	Q7XL74.pdb_B	Q6YY34	Q7XL74.pdb_B
**88**	Q7XL74.pdb_B	Q7XJV0	Q7XL74.pdb_B
**89**	Q7XL74.pdb_B	Q7XJV3	Q7XL74.pdb_B
**90**	Q7XL74.pdb_B	Q2QSQ7	Q7XL74.pdb_B
**91**	Q7XL74.pdb_B	A0A0P0V0T5	Q7XL74.pdb_B
**92**	Q7XL74.pdb_B	A2ZV04	Q7XL74.pdb_B

	subcluster_rep	member	cluster
**93**	Q7XL74.pdb_B	Q5JL91	Q7XL74.pdb_B
**94**	Q7XL74.pdb_B	Q8S5W0	Q7XL74.pdb_B
**95**	Q7XL74.pdb_B	A0A0P0Y5Q0	Q7XL74.pdb_B
**96**	Q7XL74.pdb_B	Q0J314	Q7XL74.pdb_B
**97**	Q7XL74.pdb_B	Q6EPT4	Q7XL74.pdb_B
**98**	Q7XL74.pdb_B	Q8LMS1	Q7XL74.pdb_B

Here we have two subclusters (`subcluster_rep` column). However, one of them (`A0A0P0UZA6.pdb_B`) has only one member, and if we look at it in the PyMOL session we can see that it is very similar to the other complexes. The only difference is the HMA domain being shorter. So for our purposes it would be ok to take them all together.

We can see in the `median_scores` DataFrame the model that has the best alignments to all other structures (best representative of the cluster).

In [ ]:
```
median_scores[median_scores.cluster==cluster].sort_values(by='rmsd').head(10
```

Out[ ]:

	cluster	member	tmscore	rmsd	aligned_length	cluster_size	fraction_binde
**59**	Q7XL74.pdb_B	A0A0N7KFK3	0.89660	1.49	141.0	40.0	0.93589
**61**	Q7XL74.pdb_B	A0A0P0V0T5	0.89782	1.50	140.0	40.0	0.96000
**93**	Q7XL74.pdb_B	Q84TS2	0.90749	1.52	140.0	40.0	0.97297
**62**	Q7XL74.pdb_B	A0A0P0V126	0.90529	1.58	140.0	40.0	0.97297
**90**	Q7XL74.pdb_B	Q7XJV3	0.90317	1.62	141.0	40.0	0.94805
**84**	Q7XL74.pdb_B	Q6YY31	0.90490	1.62	141.0	40.0	0.94805
**63**	Q7XL74.pdb_B	A0A0P0VKX7	0.87889	1.74	142.0	40.0	0.93670
**85**	Q7XL74.pdb_B	Q6YY33	0.88542	1.74	142.0	40.0	0.94871
**70**	Q7XL74.pdb_B	A2ZV04	0.89069	1.74	138.0	40.0	0.97222
**86**	Q7XL74.pdb_B	Q6YY34	0.88160	1.82	142.0	40.0	0.93670

We can also select the IDs of the binder proteins in this cluster, and run them through a tool such as DAVID to perform enrichment analysis.

In [ ]:
```
members = clusters[clusters.merged_rep==cluster].member
```

In [ ]:
```
for m in members:
    print(m)
```

```
A0A0P0UZA6
Q6EPT2
Q8LN41
Q6EPT4
Q6I5G4
Q7XL74
Q84TB9
Q2QSQ7
A0A0P0WEB4
Q0J314
A2ZV04
Q5JL91
Q8S5W0
Q8LNN6
Q75J02
Q6YY34
B7E663
Q7XJV3
Q6YY33
Q8LMS1
A0A0P0XD00
Q7G2B2
A0A0N7KFK3
A0A0P0V0T5
A0A0P0VKX7
A0A0P0XFR7
A0A0P0Y5Q0
A0A0P0WB87
Q10KH9
A0A0P0V126
A3ADD6
Q6YY31
A3AYA2
Q0JBG2
Q0JCK8
Q8S181
A0A0P0VRA1
Q7XJV0
Q0IYI4
Q84TS2
```

Looking at the `examples/AVRPia/AVRPia_vs_rice_clusters/merged_clusters/cluster_Q7XL74.pdb_B/session.pse` file in PyMol, we can visualize the cluster and produce a figure like this:

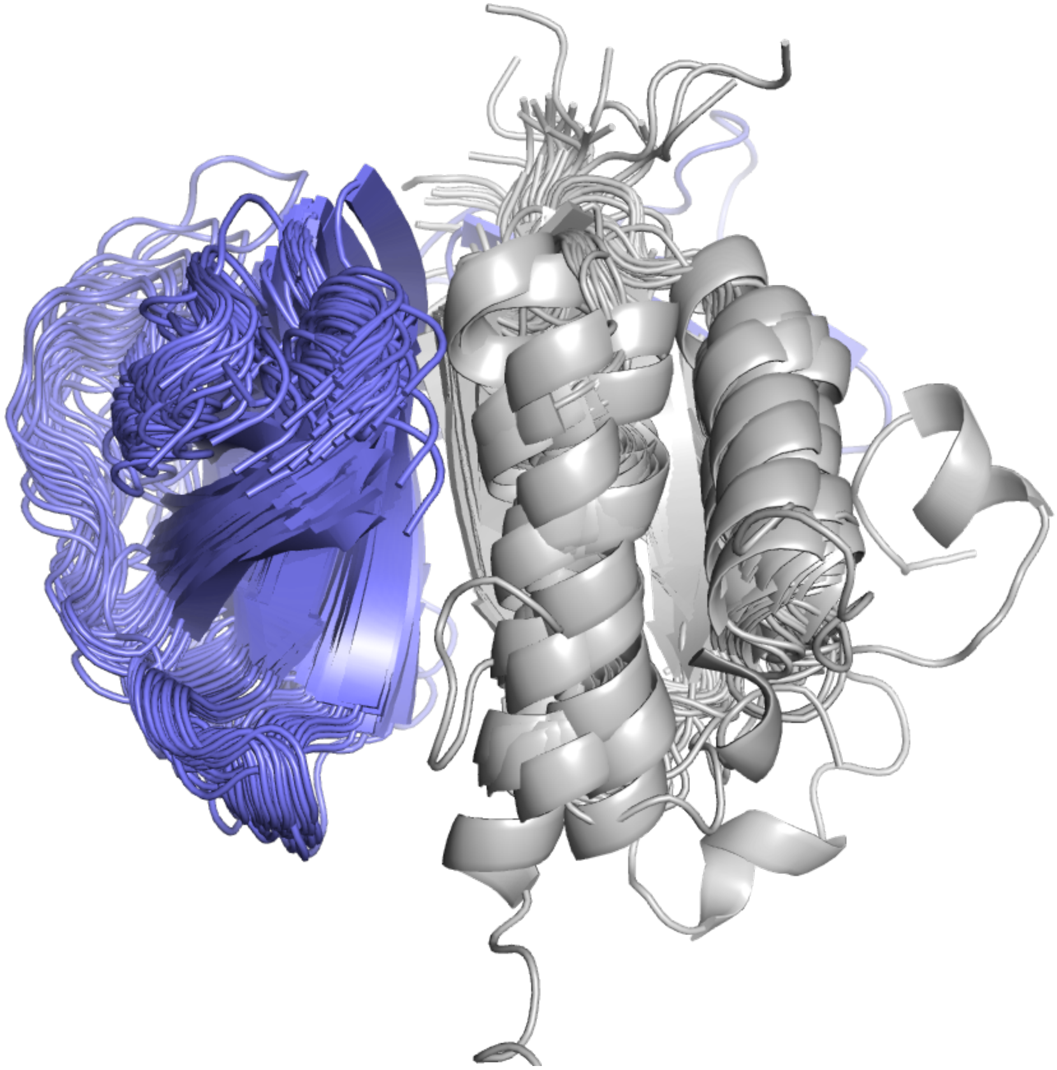

When aligning the HMA domains of the binder structures (grey), the binding of the AVR-Pia protein seems pretty consistent across most of the models in the cluster.

Tip: In PyMol, you can remove the residues with low pLDDT score to make the figure cleaner, with the command:

```
select b < 50; remove sele
```

Clustering AVR-Pik

This notebook will guide you through the process of clustering 65 complexes of the AVR-Pik protein against a sample of rice proteins. All of the models are dimeric. The 65 models originate from a proteome-wide screen of AVR-Pik against the rice proteome (O. sativa subsp. japonica, 43,000+ initial models). To select this sample we first ran AlphaCRV on all the models, and the 65 structures are part of the best clusters that were identified. Running the pipeline on these models should be enough to reproduce the results.

Prerequisites

- Install AlphaCRV on a conda environment and activate it
- Download the sequences, models and results for this example at Zenodo

1. Cluster the models with the `clustering` command

Run the following command to cluster the models. Make sure to change the paths according to your system:

```
alphacrv-cluster \
  --bait ./examples/AVRPik/AVRPik.fasta \
  --binders ./examples/AVRPik/AVRPik_binders.fasta \
  --models_dir ./examples/AVRPik/AVRPik_vs_rice_models \
  --destination ./examples/AVRPik/AVRPik_vs_rice_clusters \
  --cpus 8
```

After collecting the quality scores from the models in `--models_dir`, it will count how many models are there with an ipTM scores higher than the threshold (0.75 by default). It will prompt you to confirm or to modify the threshold. After that, it will proceed with the clustering.

The full output is as follows:

```
(env) AlphaCRV$ alphacrv-cluster \
--bait ./examples/AVRPik/AVRPik.fasta \
--binders ./examples/AVRPik/AVRPik_binders.fasta \
--models_dir ./examples/AVRPik/AVRPik_vs_rice_models \
--destination ./examples/AVRPik/AVRPik_vs_rice_clusters \
--cpus 8
INFO:root:Getting quality scores for models in
examples/AVRPik/AVRPik_vs_rice_models...
INFO:root:Found 65 model directories with quality scores.
Will select 65 models with iptm >= 0.75. Press enter to
continue, or enter a new threshold:
INFO:root:Trimming binder molecules to keep only regions with
an average PAE against the bait of up to 10.0...
INFO:root:Processed 65 complexes.
INFO:root:Writing trimmed sequences to fasta file.
INFO:root:Running sequence clustering...
INFO:root:Processing output...
INFO:root:Running structural clustering...
INFO:root:Processing output...
INFO:root:Aligning all vs all members of each cluster...
INFO:root:Aligning cluster 1 of 4...
INFO:root:11 members.
INFO:root:Aligning cluster 2 of 4...
INFO:root:21 members.
INFO:root:Aligning cluster 3 of 4...
INFO:root:13 members.
INFO:root:Aligning cluster 4 of 4...
INFO:root:20 members.
INFO:root:Calculating median alignment scores...
INFO:root:Done!!
```

After this step you will have a directory with the following structure:

```
(env) AlphaCRV$ ll examples/AVRPik/AVRPik_vs_rice_clusters/
total 24K
-rw-r--r-- 1 example g-example 1.3K Jan  3 16:30
binders_regions.csv
drwxr-xr-x 1 example g-example    0 Jan  3 16:31
merged_clusters/
drwxr-xr-x 1 example g-example    0 Jan  3 16:30
pdbs_trimmed/
drwxr-xr-x 1 example g-example    0 Jan  3 16:30 seqclusters/
drwxr-xr-x 1 example g-example    0 Jan  3 16:30 strclusters/
-rw-r--r-- 1 example g-example  19K Jan  3 16:30
trimmed_binders.fasta
```

Now let's look at some of the important files:

In [ ]:
```
from pathlib import Path
import pandas as pd
```

In [ ]:
```
results_dir = Path('./AVRPik/AVRPik_vs_rice_clusters/')
```

See clusters

The `merged_clusters.csv` file contains the list of models with their corresponding sequence, structure and merged clusters. It also has the quality scores provided by AlphaFold.

In [ ]:
```
clusters = pd.read_csv(results_dir / 'merged_clusters/merged_clusters.csv')
```

In [ ]:
```
clusters
```

Out[ ]:

	complex	str_rep	seq_rep	merged_rep	meml
**0**	6R8K-1_A0A0P0Y5A4-1	Q67VV7.pdb_B	Q6ZEZ7	Q0DEU2.pdb_B	A0A0P0Y5
**1**	6R8K-1_A0A0P0YB11-1	Q67VV7.pdb_B	Q6ZEZ7	Q0DEU2.pdb_B	A0A0P0YI
**2**	6R8K-1_A0A0P0VEM6-1	Q67VV7.pdb_B	Q6ZEZ7	Q0DEU2.pdb_B	A0A0P0VE
**3**	6R8K-1_Q8L3T8-1	Q67VV7.pdb_B	Q6ZEZ7	Q0DEU2.pdb_B	Q8L3
**4**	6R8K-1_Q2RAL3-1	Q6K9R5.pdb_B	Q6ZBC3	Q6K9R5.pdb_B	Q2RA
**...**	...	...	...	...	
**60**	6R8K-1_Q0DBF4-1	A3BDZ2.pdb_B	Q0DBF4	A3BDZ2.pdb_B	Q0DE
**61**	6R8K-1_A3ADD6-1	Q6K9R5.pdb_B	Q7G2B2	Q6K9R5.pdb_B	A3AD
**62**	6R8K-1_Q0JCK8-1	Q6K9R5.pdb_B	A0A0N7KFK3	Q6K9R5.pdb_B	Q0JC
**63**	6R8K-1_Q0JKW0-1	A0A0P0WKJ4.pdb_B	A0A0P0Y219	A0A0P0WKJ4.pdb_B	Q0JK
**64**	6R8K-1_A3BDZ2-1	A3BDZ2.pdb_B	A3BDZ2	A3BDZ2.pdb_B	A3BD

65 rows × 7 columns

The columns of the `clusters` DataFrame are:

- `complex` : The name of the complex. This is the same name as the directory where the model is stored.
- `str_rep` : Name of the structure cluster representative
- `seq_rep` : Name of the sequence cluster representative
- `merged_rep` : Name of the merged cluster representative (sequence + structure)
- `member` : The ID of the binder protein
- `iptm` : The ipTM score of the model
- `iptm+ptm` : The ipTM+PTM score of the model (it is calculated by AlphaFold as 0.8*ipTM + 0.2*pTM)

See the number of different merged clusters:

In [ ]:
```
clusters.merged_rep.unique().shape
```

Out[ ]: (4,)

For this example, the models and sequences from the 65 binder proteins were summarized in 4 clusters. Much fewer structures to sort through!

See alignment scores

Alignment scores are calcualted for each cluster by aligning all vs all members of the cluster.

In [ ]:
```
alignment_scores = pd.read_csv(results_dir / 'merged_clusters/alignment_scor
```

In [ ]:
```
alignment_scores.head()
```

Out[ ]:

	cluster	ref	member	tmscore_ref	tmscore_m	aligned_length	rms
**0**	Q0DEU2.pdb_B	A0A0P0Y5A4	A0A0P0YB11	0.88555	0.97405	432	1.4
**1**	Q0DEU2.pdb_B	A0A0P0Y5A4	A0A0P0VEM6	0.86424	0.91255	440	3.0
**2**	Q0DEU2.pdb_B	A0A0P0Y5A4	Q8L3T8	0.85603	0.93629	430	2.5
**3**	Q0DEU2.pdb_B	A0A0P0Y5A4	B9EWP2	0.86538	0.95866	425	2.1
**4**	Q0DEU2.pdb_B	A0A0P0Y5A4	Q7XV05	0.37127	0.61033	271	13.[illegible]

The columns of the `alignment_scores` DataFrame are:

- `cluster` : The name of the cluster
- `ref` : Binder ID of the reference structure in the alignment
- `member` : Binder ID of the second structure in the alignment
- `tmscore_ref` : TM-score based on the reference structure
- `tmscore_m` : TM-score based on the second structure
- `aligned_length` : Length of the alignment
- `rmsd` : RMSD of the alignment

Based on these scores, the median scores are calculated for each cluster member to find the best representative of the cluster (the one with lowest RMSD score to the other members).

Read median scores and find top clusters

Now we can rank the clusters based on the median alignment scores of the cluster representatives:

In [ ]:
```
median_scores = pd.read_csv(results_dir / 'merged_clusters/median_scores.csv
```

In [ ]:
```
median_scores.shape
```

Out[ ]:
```
(65, 7)
```

The `median_scores` DataFrame contains the median alignment scores of each cluster member when aligned to all other members of the same cluster.

In [ ]:
```
median_scores.head()
```

Out[ ]:

	cluster	member	tmscore	rmsd	aligned_length	cluster_size	fraction_
0	A0A0P0WKJ4.pdb_B	A0A0P0W913	0.43642	15.29	250.0	20.0	0.8
1	A0A0P0WKJ4.pdb_B	A0A0P0WKJ4	0.31741	13.10	219.0	20.0	0.5
2	A0A0P0WKJ4.pdb_B	A0A0P0WQF2	0.40264	12.55	226.0	20.0	0.7
3	A0A0P0WKJ4.pdb_B	A0A0P0XBZ1	0.26659	17.31	266.0	20.0	0.4
4	A0A0P0WKJ4.pdb_B	A0A0P0XUC4	0.31355	16.23	268.0	20.0	0.[illegible]

The columns of the `median_scores` DataFrame are:

- `cluster` : The name of the cluster
- `member` : ID of the cluster member (binder molecule)
- `tmscore` : Median TM-score of the complex against all other complexes in this cluster
- `rmsd` : Median RMSD of the complex against all other complexes in this cluster
- `aligned_length` : Median length of the alignment
- `cluster_size`
- `fraction_binder` : In average, how much of the binder molecule is included in the alignments of this complex against all other complexes (calculated as `(aligned_length - bait_length)/binder_length`). This is just meant to be an approximation of how complete the aligmnents are for this cluster member.

The next step is to select the cluster representatives. For this, we first need to filter out the cluster members with poor quality alignments, according to the following criteria:

- Small size
- Low median TM-score
- High median RMSD
- Low fraction of the binder aligned in the cluster representative

In [ ]:
```
# Select the clusters with the following criteria:
select = ((median_scores.cluster_size >= 5) & \
          (median_scores.tmscore >= 0.2) & \
          (median_scores.fraction_binder >= 0.2) & \
          (median_scores.rmsd <= 15))
median_scores_filtered = median_scores[select]
```

In [ ]:
```
median_scores_filtered.shape
```

Out[ ]:
```
(51, 7)
```

See how many clusters are left after filtering:

In [ ]:
```
median_scores_filtered.cluster.unique().shape
```

Out[ ]:
```
(4,)
```

Function to format tables:

In [ ]:
```
import seaborn as sns
cm_r = sns.color_palette("mako_r", as_cmap=True)
cm = sns.color_palette("mako", as_cmap=True)
```

In [ ]:
```
def make_pretty(styler):
    styler.format(precision=2)
    styler.background_gradient(axis=0, cmap=cm_r, subset=pd.IndexSlice[:,"cl
    styler.background_gradient(axis=0, cmap=cm_r, subset=pd.IndexSlice[:,"tm
    styler.background_gradient(axis=0, cmap=cm, subset=pd.IndexSlice[:,"rmsd
    styler.background_gradient(axis=0, cmap=cm_r, subset=pd.IndexSlice[:,"fr
    return styler
```

RESULT 1: See clusters ranked by RMSD

Finally, we can rank the clusters and see which ones have a good combination of low RMSD and large cluster size. These ones are the most likely to contain the true binder.

In [ ]:
```
# Select the rows with the minimum RMSD for each cluster
select = median_scores_filtered.groupby('cluster').rmsd.idxmin()
columns = ['cluster', 'tmscore', 'rmsd', 'cluster_size', 'fraction_binder']
median_scores_filtered.loc[select,columns].sort_values(by='rmsd').style.pipe
```

Out[ ]:

	cluster	tmscore	rmsd	cluster_size	fraction_binder
**41**	Q0DEU2.pdb_B	0.94	2.17	11.00	0.99
**28**	A3BDZ2.pdb_B	0.82	3.38	13.00	0.94
**54**	Q6K9R5.pdb_B	0.31	4.99	21.00	0.31
**9**	A0A0P0WKJ4.pdb_B	0.42	10.93	20.00	0.86

Here we can see that the cluster `Q0DEU2.pdb_B` has the lowest median RMSD and the highest median TM-score. However, it has a relatively small size with only 11 members.

RESULT 2: See clusters ranked by size

In [ ]:
```
# Select the rows with the minimum RMSD for each cluster
select = median_scores_filtered.groupby('cluster').rmsd.idxmin()
columns = ['cluster', 'tmscore', 'rmsd', 'cluster_size', 'fraction_binder']
median_scores_filtered.loc[select, columns].sort_values(by='cluster_size', a
```

Out[ ]:

	cluster	tmscore	rmsd	cluster_size	fraction_binder
**54**	Q6K9R5.pdb_B	0.31	4.99	21.00	0.31
**9**	A0A0P0WKJ4.pdb_B	0.42	10.93	20.00	0.86
**28**	A3BDZ2.pdb_B	0.82	3.38	13.00	0.94
**41**	Q0DEU2.pdb_B	0.94	2.17	11.00	0.99

Here we can see that the cluster `Q6K9R5.pdb_B` is the largest cluster with 21 members. It also has a low RMSD of 4.99, although the median TM-score is not the highest. This cluster, along with `A3BDZ2.pdb_B` and `A3BDZ2.pdb_B` would be good candidates for further analysis. Only three clusters with a total of 45 structures, from more than 43,000 starting models!

For this example we know that in our list of candidate binders there are 6 homologues of the true binder protein. We can find out which clusters contain these homologues:

In [ ]:
```
homologues = ['Q7XJV3', 'Q6EPT2','A0A0N7KFK3', 'Q6YY31', 'Q6EPT4', 'Q0JCK8']
```

In [ ]:
```
clusters[clusters.member.isin(homologues)][['complex','merged_rep','iptm','i
```

Out[ ]:

	complex	merged_rep	iptm	iptm+ptm
**14**	6R8K-1_Q7XJV3-1	Q6K9R5.pdb_B	0.828985	0.807492
**33**	6R8K-1_Q6EPT2-1	Q6K9R5.pdb_B	0.806888	0.763640
**36**	6R8K-1_A0A0N7KFK3-1	Q6K9R5.pdb_B	0.800443	0.782608
**45**	6R8K-1_Q6YY31-1	Q6K9R5.pdb_B	0.784981	0.775073
**56**	6R8K-1_Q6EPT4-1	Q6K9R5.pdb_B	0.766761	0.710590
**62**	6R8K-1_Q0JCK8-1	Q6K9R5.pdb_B	0.756162	0.744660

They are all in the largest cluster!

2. Make pymol sessions for the top clusters with `make_pymol_sessions`

Run the following command to select the top clusters that we saw above, make pymol sessions of the top clusters, and optionally do structural clustering on each cluster to find subclusters:

```
alphacrv-rank \
  --clusters_dir /path/to/destination/6R8K_clusters \
  --min_members 5 \
  --min_tmscore 0.2 \
  --max_rmsd 15 \
  --cluster_clusters
```

The program will show you the top clusters that will be used to make the pymol sessions. You can press `Enter` to continue, or exit the program with `Ctrl+C` to change the filtering parameters.

```
(env) AlphaCRV$ alphacrv-rank \
--clusters_dir ./examples/AVRPik/AVRPik_vs_rice_clusters \
--min_members 5 \
--min_tmscore 0.2 \
--max_rmsd 15 \
--cluster_clusters
INFO:root:Identified 4 top clusters.
INFO:root:Top clusters:

            cluster   tmscore    rmsd  cluster_size
fraction_binder
0      Q6K9R5.pdb_B  0.313905   4.985          21.0
0.305328
1  A0A0P0WKJ4.pdb_B  0.421400  10.930          20.0
0.856436
2      A3BDZ2.pdb_B  0.824705   3.385          13.0
0.944444
3      Q0DEU2.pdb_B  0.940945   2.165          11.0
0.987633

Press Enter to continue, or Ctrl+C to exit and select
different filtering parameters:
INFO:root:Copying pdbs from the top clusters...
INFO:root:Making Pymol sessions...
PyMOL>select chain B AND model A0A0P0WKJ4_repB
 Selector: selection "sele" defined with 3937 atoms.
PyMOL>bg white

...
PyMOL>select chain B AND model Q6K9R5_repB
 Selector: selection "sele" defined with 1397 atoms.
PyMOL>bg white
PyMOL>set ray_shadow, 0
 Setting: ray_shadow set to off.
PyMOL>color grey80
 Executive: Colored 61825 atoms.
PyMOL>select chain A
 Selector: selection "sele" defined with 31164 atoms.
PyMOL>color slate, sele
 Executive: Colored 31164 atoms.
PyMOL>delete all
INFO:root:Clustering clusters...
INFO:root:Clustering A0A0P0WKJ4.pdb_B
INFO:root:Running structural clustering...
INFO:root:Processing output...
INFO:root:Clustering A3BDZ2.pdb_B
INFO:root:Running structural clustering...
INFO:root:Processing output...
INFO:root:Clustering Q0DEU2.pdb_B
INFO:root:Running structural clustering...
INFO:root:Processing output...
```

```
INFO:root:Clustering Q6K9R5.pdb_B
INFO:root:Running structural clustering...
INFO:root:Processing output...
INFO:root:Done!!
```

This command should create the following files in the `--clusters_dir` / `merged_clusters` directory:

- `clustered_clusters.csv` : Contains the subclusters for each of the top clusters.
- `cluster_<cluster_ID>/` : Contains the PDBs of each cluster, and a PyMol session with the cluster members.
- `cluster_<cluster_ID>_clusters/` : Contains the results of the `foldseek easy-cluster` run on the cluster members.

Read clustered clusters

The following DataFrame contains the subclusters for each of the top clusters:

In [ ]:
```
clustered_clusters = pd.read_csv(results_dir / 'merged_clusters/clustered_cl
```

In [ ]:
```
clustered_clusters.head()
```

Out[ ]:

	subcluster_rep	member	cluster
**0**	A0A0P0W913.pdb_B	A0A0P0W913	A0A0P0WKJ4.pdb_B
**1**	A0A0P0W913.pdb_B	A0A0P0WQF2	A0A0P0WKJ4.pdb_B
**2**	A0A0P0WKJ4.pdb_B	A0A0P0WKJ4	A0A0P0WKJ4.pdb_B
**3**	A0A0P0WKJ4.pdb_B	Q0JKW0	A0A0P0WKJ4.pdb_B
**4**	A0A0P0XBZ1.pdb_B	A0A0P0XBZ1	A0A0P0WKJ4.pdb_B

Now we can look at the most interesting clusters and their subclusters in more detail:

RESULT 1: Cluster Q6K9R5.pdb_B (contains true binder homologs, top cluster by size)

In [ ]:
```
cluster = 'Q6K9R5.pdb_B'
```

See subclusters:

In [ ]:
```
clustered_clusters[clustered_clusters.cluster==cluster]
```

Out[ ]:

	subcluster_rep	member	cluster
**44**	Q6K9R5.pdb_B	Q6K9R5	Q6K9R5.pdb_B
**45**	Q6K9R5.pdb_B	A0A0N7KFK3	Q6K9R5.pdb_B
**46**	Q6K9R5.pdb_B	B7E663	Q6K9R5.pdb_B
**47**	Q6K9R5.pdb_B	Q6EPT2	Q6K9R5.pdb_B
**48**	Q6K9R5.pdb_B	Q7XJV3	Q6K9R5.pdb_B
**49**	Q6K9R5.pdb_B	Q6YY31	Q6K9R5.pdb_B
**50**	Q6K9R5.pdb_B	Q7G2B2	Q6K9R5.pdb_B
**51**	Q6K9R5.pdb_B	A3ADD6	Q6K9R5.pdb_B
**52**	Q6K9R5.pdb_B	Q94CS5	Q6K9R5.pdb_B
**53**	Q6K9R5.pdb_B	Q0JCK8	Q6K9R5.pdb_B
**54**	Q6K9R5.pdb_B	Q6ZBC3	Q6K9R5.pdb_B
**55**	Q6K9R5.pdb_B	Q2RAL3	Q6K9R5.pdb_B
**56**	Q6K9R5.pdb_B	Q5JL91	Q6K9R5.pdb_B
**57**	Q6K9R5.pdb_B	Q84TB9	Q6K9R5.pdb_B
**58**	Q6K9R5.pdb_B	A0A0P0XYR1	Q6K9R5.pdb_B
**59**	Q6K9R5.pdb_B	Q10N90	Q6K9R5.pdb_B
**60**	Q6K9R5.pdb_B	Q2QZ01	Q6K9R5.pdb_B
**61**	Q6K9R5.pdb_B	Q7EY69	Q6K9R5.pdb_B
**62**	Q6K9R5.pdb_B	Q6EPT4	Q6K9R5.pdb_B
**63**	Q6K9R5.pdb_B	Q8L4I2	Q6K9R5.pdb_B
**64**	Q6K9R5.pdb_B	Q8LJL3	Q6K9R5.pdb_B

Only one subcluster (`subcluster_rep` column).

We can see in the `median_scores` DataFrame the model that has the best alignments to all other structures (best representative of the cluster).

In [ ]:

```
median_scores[median_scores.cluster==cluster].sort_values(by='rmsd').head(10
```

Out[ ]:

	cluster	member	tmscore	rmsd	aligned_length	cluster_size	fraction_binc
**54**	Q6K9R5.pdb_B	Q6EPT4	0.313905	4.985	167.5	21.0	0.3053
**51**	Q6K9R5.pdb_B	Q2RAL3	0.772890	5.370	163.0	21.0	0.9722
**57**	Q6K9R5.pdb_B	Q6ZBC3	0.591925	5.620	161.0	21.0	0.9315
**61**	Q6K9R5.pdb_B	Q84TB9	0.608545	6.680	162.0	21.0	0.9718
**45**	Q6K9R5.pdb_B	A0A0P0XYR1	0.548765	7.355	142.0	21.0	0.5903
**50**	Q6K9R5.pdb_B	Q2QZ01	0.545625	7.420	142.0	21.0	0.5903
**53**	Q6K9R5.pdb_B	Q6EPT2	0.575325	7.915	167.0	21.0	0.8915
**58**	Q6K9R5.pdb_B	Q7EY69	0.544055	8.060	132.0	21.0	0.4698
**49**	Q6K9R5.pdb_B	Q10N90	0.553010	8.125	139.0	21.0	0.5542
**64**	Q6K9R5.pdb_B	Q94CS5	0.604640	8.180	163.0	21.0	0.9722

We can also select the IDs of the binder proteins in this cluster, and run them through a tool such as DAVID to perform enrichment analysis.

In [ ]:
```
members = clusters[clusters.merged_rep==cluster].member
```

In [ ]:
```
for m in members:
    print(m)
```

```
Q2RAL3
B7E663
Q7XJV3
Q94CS5
Q6ZBC3
Q8LJL3
Q10N90
Q8L4I2
Q5JL91
Q7EY69
Q6EPT2
A0A0N7KFK3
Q84TB9
A0A0P0XYR1
Q2QZ01
Q6YY31
Q7G2B2
Q6EPT4
Q6K9R5
A3ADD6
Q0JCK8
```

Looking at the `examples/AVRPik/AVRPik_vs_rice_clusters/merged_clusters/cluster_Q6K9R5.pdb_B/session.pse` file in PyMol, we can visualize the cluster and produce a figure like this:

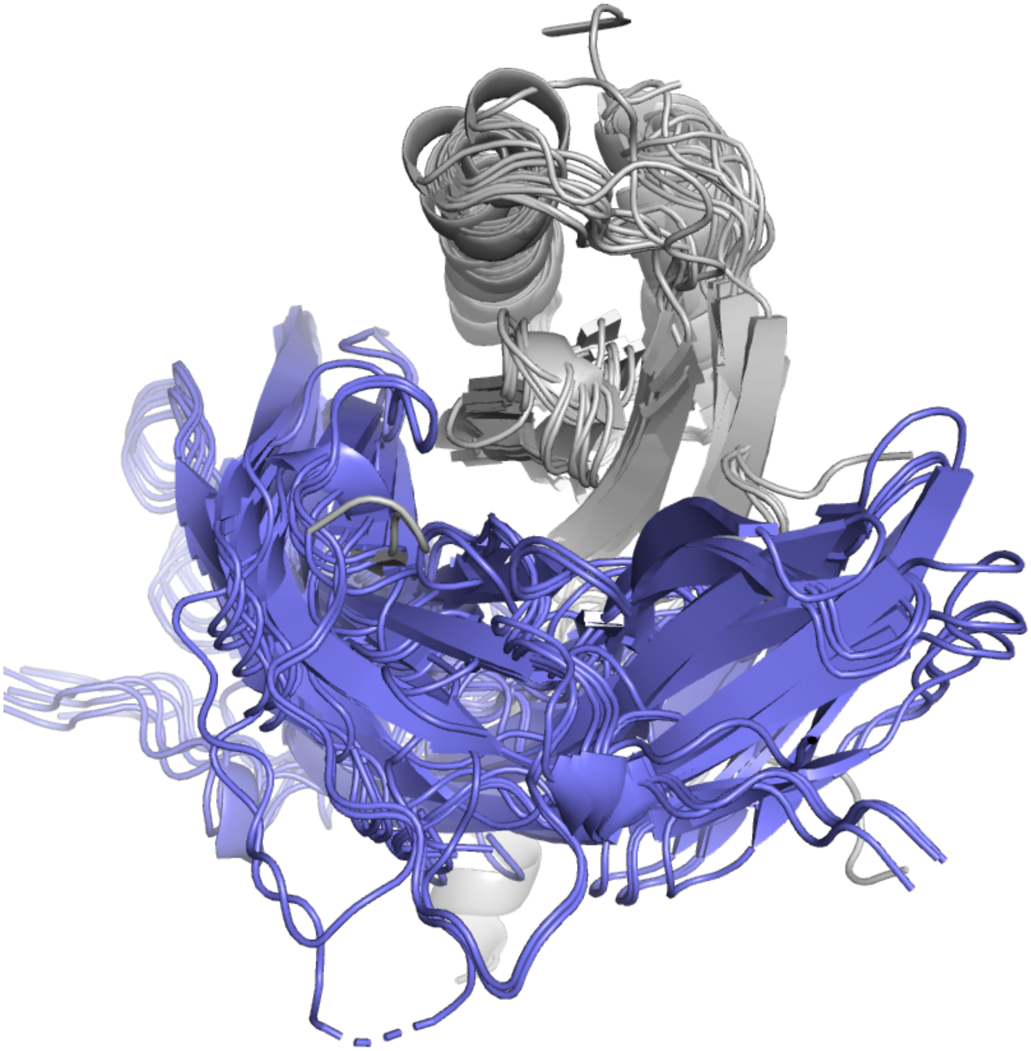

When aligning the HMA domains of the binder structures (grey), the different locations of the AVR-Pik protein raise the posibility of a more flexible binding across the entire surface of the HMA domain's beta strand.

Tip: In PyMol, you can remove the residues with low pLDDT score to make the figure cleaner, with the command:

```
select b < 50; remove sele
```
